# Intermolecular base stacking mediates RNA-RNA interaction in a crystal structure of the RNA chaperone Hfq

**DOI:** 10.1038/s41598-017-10085-8

**Published:** 2017-08-29

**Authors:** Eike C. Schulz, Markus Seiler, Cecilia Zuliani, Franka Voigt, Vladimir Rybin, Vivian Pogenberg, Norbert Mücke, Matthias Wilmanns, Toby J. Gibson, Orsolya Barabas

**Affiliations:** 1 Structural and Computational Biology Unit, European Molecular Biology Laboratory, 69117 Heidelberg, Germany; 2 Hamburg Outstation, European Molecular Biology Laboratory, Hamburg, 22603 Germany; 3 Protein Expression and Purification Core Facility, European Molecular Biology Laboratory, 69117 Heidelberg, Germany; 4 Division Biophysics of Macromolecules, German Cancer Research Center, Heidelberg, 69120 Germany; 5 Present Address: Max Planck Institute for the Structure and Dynamics of Matter, Luruper Chaussee 149, 22761 Hamburg, Germany; 6 Buchmann Institute for Molecular Life Sciences, Max-von-Laue-Str. 15, 60438 Frankfurt a.M., Germany; 7 Present Address: Friedrich Miescher Institute for Biomedical Research, Maulbeerstrasse 66, 4058 Basel, Switzerland

## Abstract

The RNA-chaperone Hfq catalyses the annealing of bacterial small RNAs (sRNAs) with target mRNAs to regulate gene expression in response to environmental stimuli. Hfq acts on a diverse set of sRNA-mRNA pairs using a variety of different molecular mechanisms. Here, we present an unusual crystal structure showing two Hfq-RNA complexes interacting via their bound RNA molecules. The structure contains two Hfq_6_:A_18_ RNA assemblies positioned face-to-face, with the RNA molecules turned towards each other and connected via interdigitating base stacking interactions at the center. Biochemical data further confirm the observed interaction, and indicate that RNA-mediated contacts occur between Hfq-RNA complexes with various (ARN)_X_ motif containing RNA sequences *in vitro*, including the stress response regulator OxyS and its target, *fhlA*. A systematic computational survey also shows that phylogenetically conserved (ARN)_X_ motifs are present in a subset of sRNAs, some of which share similar modular architectures. We hypothesise that Hfq can co-opt RNA-RNA base stacking, an unanticipated structural trick, to promote the interaction of (ARN)_X_ motif containing sRNAs with target mRNAs on a “speed-dating” fashion, thereby supporting their regulatory function.

## Introduction

Non-coding RNAs play key roles in regulating gene expression in all domains of life. In bacteria, sRNAs control almost every aspect of bacterial physiology including metabolism, quorum sensing, and virulence^1–4^. During stress and environmental changes, sRNAs orchestrate a complex and dynamic response, allowing the bacteria to rapidly adapt to new conditions. Thus, they play critical roles in the lifestyle switching of bacteria that are able to inhabit variable environments, as well as during infection and disease^5–10^.

Bacterial sRNAs are ~50–300 nucleotides long, and act by modulating the stability and translation of diverse mRNAs. They are expressed mostly independently from their mRNA targets and many of them can simultaneously act on several different mRNAs^11–13^, regulating the translation of all of these with specificity and precision. Target recognition was shown to be generally initiated at short complementary ‘seed’ regions in the two RNAs^14, 15^ and for most sRNA-mRNA pairs it is critically dependent on the RNA-chaperoning protein Hfq^7, 16–19^. Consequently, bacteria with mutations in the *hfq* gene show reduced virulence and reduced adaptation potential^16, 18^.

The Hfq protein is a homo-hexameric ring-shaped RNA-binding protein of the Sm/LSm family that has several distinct ways of interacting with RNA^20–22^. In sRNAs, Hfq was shown to preferentially bind 3′ to seed regions, whereas it interacts 5′ to sRNA-target regions in mRNAs^23^. Furthermore, Rho-independent terminators display a universal recognition motif for Hfq^23^. The Hfq hexamer (Hfq_6_) has three distinct RNA binding sites referred to as ‘proximal’, ‘distal’ and ‘lateral’ (reviewed in refs 19, 22 and 24). In addition, its flexible C-terminal tail can contribute to binding and regulation of some RNAs^25–28^. The ‘lateral’ binding site is located on the rim of the Hfq_6_ ring and has accessory roles in RNA binding, with a preference for UA-rich sequences^27, 29–31^. The ‘proximal’ site on one face of the ring preferentially binds to U-rich RNA sequences, such as the poly(U) tracts present at the 3′ termini of most sRNAs^32, 33^. At these poly(U) tails, Hfq also directly interacts with the free 3′-OH group, which helps trigger a constricted RNA conformation required for efficient sRNA binding and recognition^32^. The ‘distal’ site is located on the opposite face of the ring and has high affinity to A-rich sequences, which are commonly found in the 5′ untranslated regions (UTR) of mRNAs^19, 34^. Crystal structures revealed that the distal site of each Hfq subunit can accommodate a triplet of RNA nucleotides (ARN or AAN) with differing specificities: the A-site binds specifically adenines, the R-site can accommodate both adenine and guanine with preference for A, while the third base points away from Hfq towards the solvent and can be any nucleotide (N)^26, 35^. Six such sites come together in the hexamer to form a circular binding site accommodating an 18nt long A-rich RNA segment^36^. In agreement, genomic SELEX experiments revealed a specific enrichment of A-rich sequences among Hfq-bound RNAs and *in vivo* UV-crosslinking demonstrated that Hfq specifically binds to repeated ARN triplets (referred to as (ARN)_X_ motifs) in the 5′-UTR of mRNAs^34, 37^.

However, Hfq-RNA binding is not restricted to a single binding site. Recent reports indicate that Hfq-RNA interactions can simultaneously involve multiple sites on the RNA and/or the protein^21, 30, 38, 39^. A remarkable example is demonstrated in the crystal structure of the Hfq-RydC complex. Here, the 3′ U-rich tail of the sRNA binds to the proximal face of Hfq, while the 5′ end binds to the lateral surface. In addition, the external part of the rim as well as Hfq’s intrinsically disordered C-terminal tail are involved in contacts with RydC^27^. Furthermore, whereas most sRNAs primarily bind to Hfq’s proximal site, some also contain (ARN)_X_-like motifs, which contribute to their stability and can bind to the distal site of Hfq^21, 40–43^. One prominent example is the oxidative stress response regulator, OxyS that is induced upon oxidative stress and acts on multiple mRNAs to fine-tune the expression of various stress response pathways^5, 44, 45^. In addition to proximal site binding regions^39, 43^, OxyS contains an extended (ARN)_X_ motif (positions 59–86) that is essential for its regulatory function, and biochemical studies and crystal structures have shown that it binds to Hfq’s distal site^42, 46^.

To catalyse the annealing of diverse sets of RNA pairs, Hfq has been shown to employ a variety of mechanistic strategies (reviewed in refs 19, 24 and 47). For example, Hfq binding can reduce RNA motility and flexibility, which increases the chance for two RNA molecules to meet and the on-rate of their interaction. Hfq can also alter RNA secondary structure and thereby expose complementary regions in sRNAs and mRNAs, enabling their pairing or helping to form more stable s/mRNA pairs compared to the ones formed spontaneously. In addition, the distinct specificities of Hfq’s proximal and distal binding sites allow sRNAs and mRNAs to bind simultaneously to opposite faces of a single Hfq hexamer, which increases their local concentration and facilitates annealing. Arginine-rich patches along the rim of the protein are proposed to guide and catalyse base pairing between complementary strands^29, 30^. Moreover, the repetitive binding surfaces of the Hfq hexamer can accommodate multiple RNA molecules on the same surface, which was proposed to enable cycling of different RNA substrates on the ring and facilitate RNA release and turnover^48^. RNA turnover is further supported by Hfq’s C-terminal tail that helps displace RNA duplexes from the core binding sites^49^. Finally, Hfq can also interact with various proteins involved in RNA metabolism and translation, which help to mediate its function^50, 51^. It appears that Hfq uses different mechanisms to catalyse annealing depending on the exact sRNA-mRNA pair, and the variety of the documented, partially complementary, mechanistic pathways enables this global ribo-regulator to act on many sRNA substrates and mRNA targets rapidly and accurately in the crowded milieu of the cell^41, 48, 50–54^. Nevertheless, the exact mechanisms of pairing remain incompletely understood for many sRNA-mRNA target pairs.

Here, we present a crystal structure of *Escherichia coli* Hfq in complex with A_18_ RNA that shows an unanticipated quaternary architecture with two Hfq_6_:A_18_ assemblies interacting via their RNA molecules. Remarkably, the RNA molecules are held together by base stacking of every third base, the N bases of the (ARN)_X_ motif, that are flipped out by Hfq. Consistent with the structure, biochemical data with RNA probes that lack the base at the N-site and a systematic computational survey support the notion that base stacking of the N-site bases can help mediate RNA-RNA interaction between Hfq-bound (ARN)_X_ motif-containing RNA molecules. We hypothesize that Hfq co-opts the N-site bases to initiate low-affinity interactions between RNA substrates so as to facilitate their partner search, adding yet another tool to the toolbox of this versatile RNA chaperone.

## Results

### Crystal structure of an Hfq-A_18_ RNA complex shows base stacking between two Hfq-bound RNA molecules

**Figure 1:**
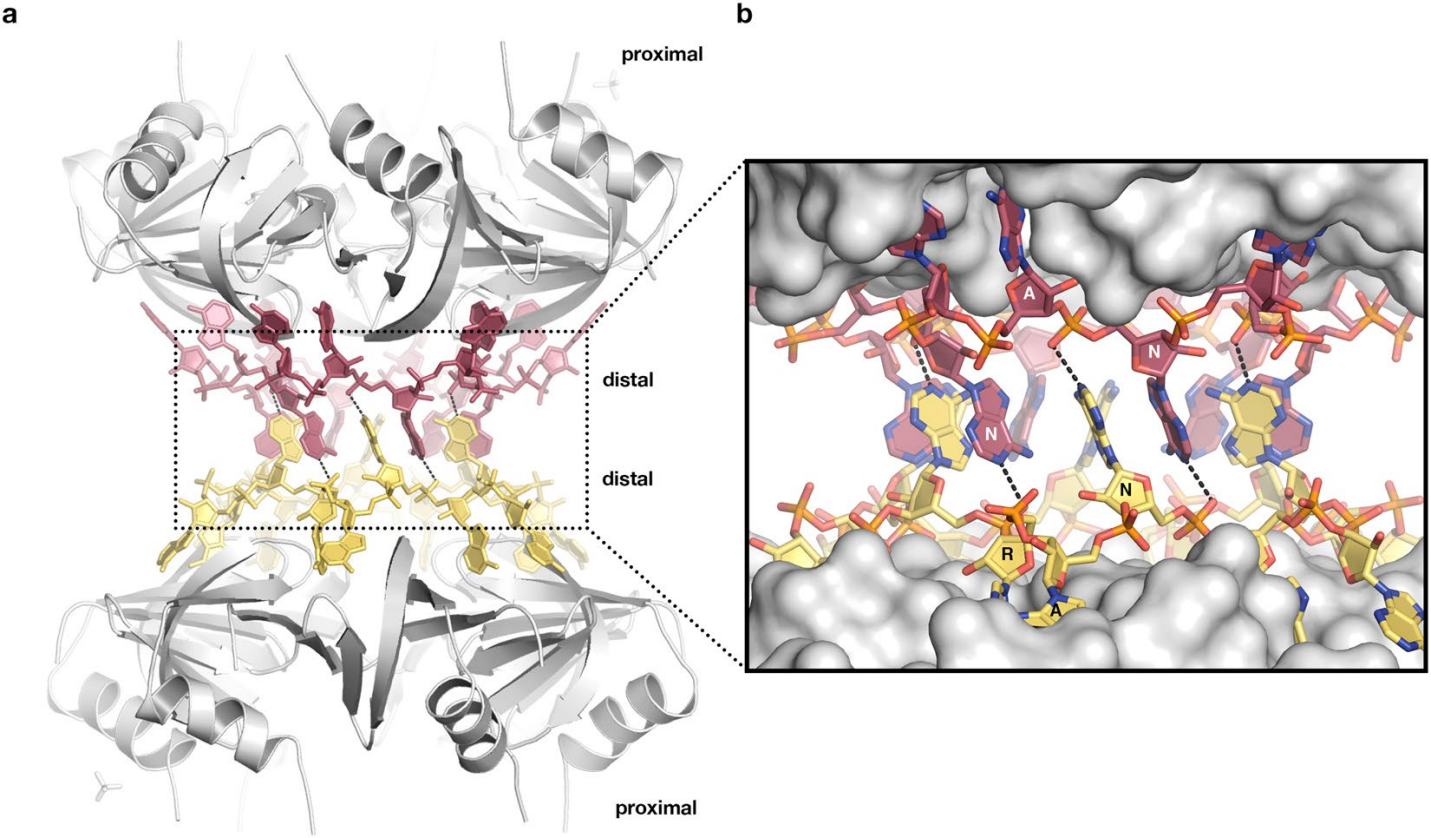
Crystal structure of an Hfq-poly(A) complex reveals interaction via base stacking of the RNA rings. (**a**) Overall view of the *E*. *coli* Hfq72-A_18_ crystal structure showing a sandwich-like dimeric assembly of two RNA-bound Hfq hexamers. The two Hfq rings (grey) comprise amino acids 1–72 and are arranged with their distal faces oriented towards each other; the RNA chains (dark red/yellow) are located at the center between the proteins and form interlocked base stacking interactions holding the assembly together. (**b**) Close-up of the RNA interactions showing the A-site and R-site bases docked deep into Hfq’s surface and the flipped-out N-site bases interacting via base stacking and electrostatic interactions (dashed lines).

Several crystal structures of Hfq have been described previously alone or in complex with various RNA substrates^22, 27, 32, 35, 46, 55, 56^. These data revealed how Hfq recognizes various RNA molecules and suggested mechanistic models for their annealing, however the structural basis of Hfq-mediated RNA-RNA interaction remains incompletely understood. Here, we present the crystal structure of an Hfq_6_-A_18_ RNA complex at 2.5 Å resolution (Fig. 1 and Table 1) that reveals an unanticipated quaternary structure. The crystals resulted from an experiment aimed at co-crystallizing *Escherichia coli* Hfq72 (containing amino acids 1–72) with A_30_ RNA and poly(A)-polymerase 1, but they contain only Hfq72 and an 18 nucleotide long poly(A) RNA segment. Hfq72, that lacks most of the intrinsically disordered C-terminal tail^25, 57^, was used to facilitate crystallization. Recent data showed that deletion of the highly variable C-terminus^25, 58^ has no effect on the affinity or annealing of A_18_-containing RNAs and indicate that its main function is to promote RNA turnover^49^. In the resulting crystal structure, the protein itself looks very similar to previously published structures^35, 55^ and only small changes can be observed (Figure S1a). Consistent with previous reports^55, 59^, both the proximal and the distal sites of the Hfq72 hexamer are occupied with RNA (Figure S1b,c). The electron density at the proximal site is weak, probably indicating partial occupancy (Figure S1c). While this made the identification of the bases ambiguous, they were interpreted as uridines because they exhibit the shape of pyrimidine bases and Hfq is known to preferentially bind U-rich RNA at this site. Since no uridine containing RNA or nucleotides were added in the crystallization experiments, this density probably originated from the cellular lysate or from contamination in the synthetic RNA samples. At the distal face, we detect strong electron density for the A_18_ RNA segment (Figure S1b), whereas the remaining 12 nts of the A_30_ RNA substrate are not visible. The A_18_ chain adopts a very similar binding geometry as previously reported, with most nucleotides in the C2′-endo configuration, the A- and R-site bases tightly bound to the surface of Hfq, and the N-site bases pointing away from the protein surface^35^. However, unlike in previous structures, the N-site bases are not freely exposed to the solvent, instead they form interdigitating base stacking interactions (ring-to-ring distances ~3.8 Å) with a neighbouring Hfq72_6_:A_18_ complex resulting in a (Hfq72_6_:A_18_)_2_ dimer (Fig. 1). In this sandwich-shaped supramolecular assembly, two A_18_ RNA molecules are enclosed between two hexameric Hfq72 protein rings and the stacking of the N-site bases provides the glue to hold the assembly together. With respect to other Hfq-poly(A) structures, the N-site adenines are tilted only slightly - approximately by 15 degrees - towards the surface of Hfq (Figure S1a), and their stacking do not induce significant conformational changes. In addition to the base stacking, the N_1_ atom of each N-site adenine makes an electrostatic interaction with a phosphate group (distance to O_2P_ 2.7 Å) in the sugar-phosphate backbone of the A_18_ chain of the partner Hfq72_6_:A_18_ ring (Fig. 1b). Considering the low pH (4.2) of the crystallization solution, it is possible that the adenine base is protonated or tautomerized in the crystals, allowing a proper hydrogen bond to form between its N_1_ and the phosphate oxygen of the partner RNA. These interactions stabilize the conformation of the stacked bases and the dimeric assembly. There are no direct protein-protein interactions between the Hfq72 hexamers and the dimer is held together solely by RNA-RNA stacking.

**Table 1:** X-ray Data Collection and Refinement Statistics.

Data collection		Refinement	
Wavelength (Å)	0.979681	Resolution limits (Å)	40.60–2.51
Cell dimensions (Å)		No. reflections	6816
a	66.88	No. atoms	
b	66.88	macromolecules	1192
c	227.72	ligands	25
α	90.0	water	44
β	90.0	R_work_ (%)/R_free_ (%)	19.64/25.22
γ	120.0		
Space group	R32	B-factors	
Resolution range (Å)	40.60–2.51 (2.60–2.51)	macromolecules	36.5
No. reflections	31502 (1689)	water	39.8
I/σ	15.03 (3.18)		
Completeness (%)	96.8 (70.1)	R.m.s. deviations	
Redundancy	4.6 (3.0)	Bond lengths (Å)	0.009
R_meas_ (%)	7.7 (43.8)	Bond angles (°)	1.095

Values in parentheses indicate the specific values in the highest resolution shell. R_meas_ is defined in detail in ref. 99.

### Base stacking between Hfq-RNA complexes in solution

**Figure 2:**
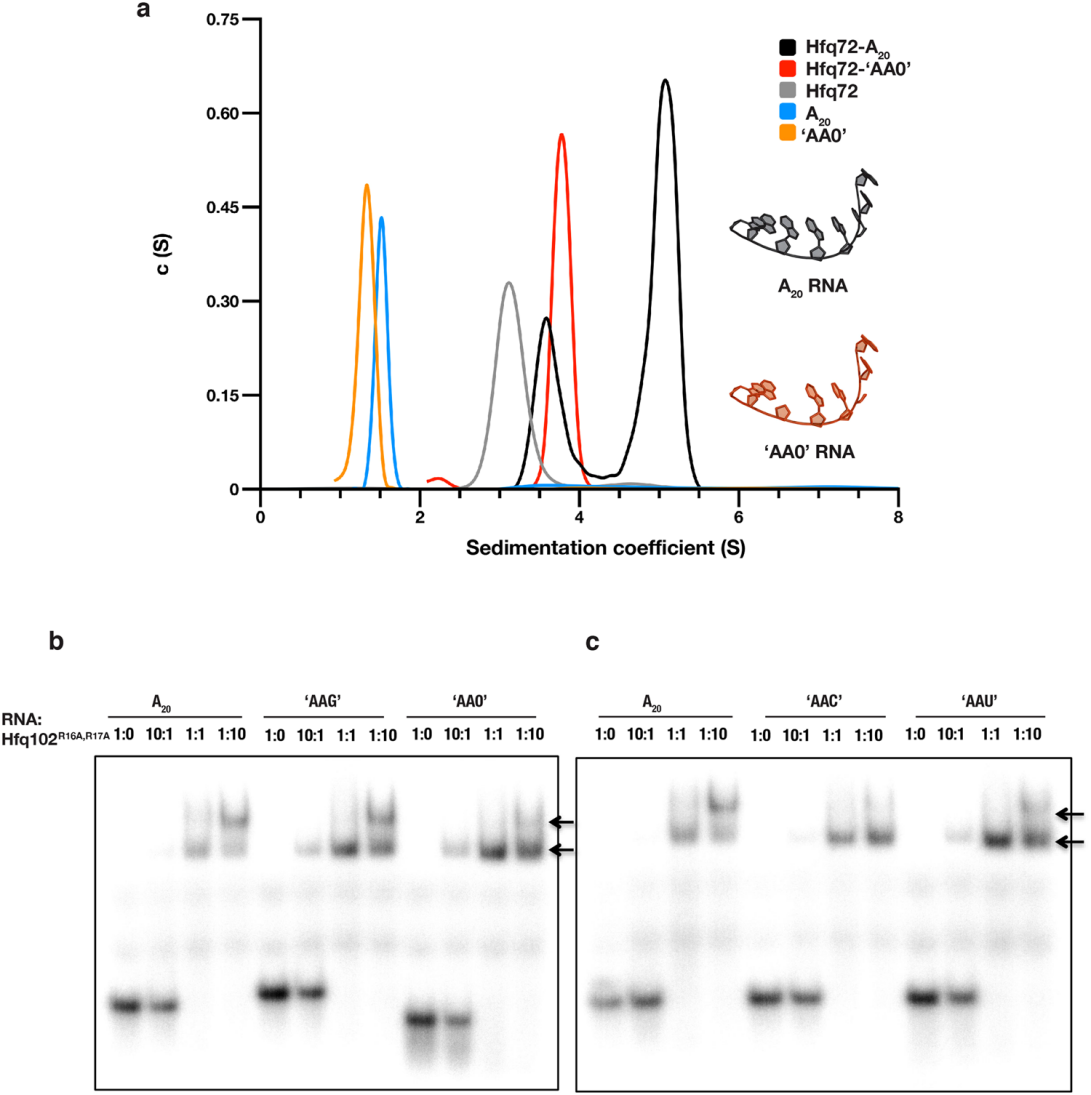
Hfq-A_20_ complexes form RNA-mediated supramolecular dimers in solution. **(a)** Sedimentation velocity curves of various Hfq72-RNA complexes. All individual components (Hfq72, A_20_, and ‘AA0’) sediment corresponding to their expected sedimentation coefficients, the Hfq72-‘AA0’ complex shows a peak consistent with a single Hfq72_6_:‘AA0’ ring, while the Hfq72-A_20_ curve reveals a faster sedimenting, larger assembly. The apparent sedimentation coefficient of this peak is between the expected values of stable Hfq72_6_:A_20_ monomers and dimers, implying a dynamic equilibrium between the two assemblies. The additional small peak corresponds to excess Hfq72. (**b**,**c**) Electrophoretic mobility shift assays showing two distinct complexes (arrows) on native PAGE gels. 20 nM radioactively (5′-^32^P) labelled ssRNA was incubated with increasing amounts of Hfq102^R16A,R17A^ (2 nM–200 nM). A higher-order complex is observed in the Hfq102^R16A,R17A^-A_20_ and Hfq102^R16A,R17A^-‘AAG’ (**b**) samples, whereas greatly reduced in the Hfq102^R16A,R17A^-‘AA0’ (**b**), Hfq102^R16A,R17A^-‘AAC’ and Hfq102^R16A,R17A^-‘AAU’ samples (**c**).

To explore if the base stacking mediated dimerization observed in the crystal structure also occurs in solution, we analysed the oligomeric state of Hfq72-A_20_ complexes by analytical ultracentrifugation (AUC). This revealed a shift in the sedimentation coefficient (s = 5.1S) compared to the RNA-free Hfq72 control (s = 3.1S), indicating that larger molecular assemblies have formed. Notably, the shift was significantly larger than expected for a simple monomeric Hfq72_6_:A_20_ complex (s = 3.9S, calculated based on our crystal structure) (Fig. 2a and Table S1) and its exact value was dependent on the concentration; it approached the values calculated for Hfq72_6_:A_20_ monomers at low complex concentrations, but increased gradually with increasing concentration (data not shown). These results indicate that Hfq72_6_:A_20_ complexes can form dimeric assemblies. The reduced s-value probably indicates a dynamic equilibrium with the monomeric species. Such dynamic oligomerization equilibrium would result in average sedimentation coefficients that are between the predicted s-values of monomers and dimers and increase with the abundance of the larger assemblies as concentration increases^60^.

While AUC can provide information about the relative size of the Hfq-RNA complex, it does not reveal their intermolecular arrangement. Thus, to determine if the observed higher order complexes are arranged face to face, held together by the flipped-out N-site bases of the RNA molecules as in the crystal structure, we tested how the removal of these bases affects oligomerization in AUC. We used a synthetic A_20_ RNA derivative, which contained an intact sugar-phosphate backbone, but every third nucleotide (the N-site equivalent) was substituted with an abasic nucleotide (‘AA0’). This ‘AA0’ RNA was able to bind to Hfq72 equally well as A_20_ (Figures S2–3), consistent with previous observations that the N-site bases do not contribute to Hfq binding^35^. On the other hand, removal of the N-site bases abolished formation of supramolecular assemblies in AUC experiments: the Hfq72-‘AA0’ complex sedimented as a single specie with a sedimentation coefficient consistent with a monomeric Hfq72_6_:‘AA0’ assembly (measured s = 3.8 S to be compared with the expected s = 3.9 S), and no shift to larger assemblies could be observed (Fig. 2a and Table S1).

Next, to further confirm Hfq_6_:RNA dimerization and its dependence on the N-site bases, we performed isothermal titration calorimetry (ITC, Figure S3) and fluorescence anisotropy (FA, Figure S4) experiments. These revealed a single binding event for the Hfq72-‘AA0’ RNA interaction, while showing two consecutive binding events with A_20_ RNA. The binding affinities measured for the first event (K_D1-ITC_ = 1.3 nM and K_D1-FA_ = 0.4 nM) are consistent with previously reported Hfq-poly(A) binding constants, as well as with the single binding constant measured for the ‘AA0’ RNA (K_D-ITC_ = 40 nM)^35^. In contrast, the second binding has lower affinity (K_D2-ITC_ = K_D2-FA_ = 2.2 µM) and is only observed with A_20_ but not with ‘AA0’. This implies that the first high-affinity association event corresponds to primary Hfq-RNA binding, while the second A_20_-specific moderate-affinity event may represent Hfq-A_20_ dimerization. The moderate dimerization affinity observed with the Hfq72_6_:A_20_ complex is also consistent with our size-exclusion data where the micro-molar affinity dimers cannot be observed (Figure S2), and with our AUC data showing a sedimentation coefficient slightly smaller than expected for dimers (as described above)^60^.

We confirmed these results using electrophoretic mobility shift assays (EMSA) with full length Hfq (Hfq102). To avoid protein aggregation in EMSA, we used the Hfq102^R16A,R17A^ mutant^30, 61, 62^. These experiments revealed two shifted bands with the A_20_ RNA, one likely corresponding to monomeric Hfq102^R16A,R17A^_6_:A_20_ complexes and the second to a slower migrating larger species. Consistent with the AUC, ITC and FA data, the slower migrating (‘super-shifted’) band was greatly reduced in the Hfq102^R16A,R17A^-‘AA0’ complexes (Fig. 2b).

Finally, to further explore the impact of the N-site bases on Hfq:RNA oligomerization, we performed EMSA experiments with A_20_ variants, where every N-site base was replaced with G, C or U (‘AAG’, ‘AAC’ and ‘AAU’ derivative). Since base stacking can occur with any base, we predicted that dimers can form with diverse RNA sequences, but their affinity might differ depending on the base stacking efficiencies of different bases^63^. Consistently, we observed significant amount of supershift with ‘AAG’ that contains strongly stacking purine bases at the N-sites, but detected smaller amount of larger assemblies with pyrimidine bases as in ‘AAC’ and ‘AAU’ (Fig. 2b,c). Interestingly, the supershifted band was practically absent with C at the N-site, consistent with its lowest base stacking efficiency^63^. The observed selectivity might also be supported by electrostatic or hydrogen bonding interaction between the base and the phosphate group of the partner RNA as seen in our crystal structure: guanine can naturally form a strong hydrogen bond at the N_1_ position, whereas pyrimidines might not suitably reach the partner phosphate backbone.

Together, these data indicate that RNA-mediated Hfq-RNA dimers form in solution and their assembly requires the flipped-out N-site bases. While the biophysical data cannot directly reveal the exact architecture of the detected supramolecular assemblies, the results are in perfect agreement with our crystal structure. Especially, the peculiar dependence of the interaction on the N-site bases is uniquely explained by the structural data, whereas absence of these bases would not be expected to affect other Hfq-RNA assemblies.

### Base stacking brings together (ARN)_X_ motifs from OxyS and *fhlA*

Our structural and biochemical data imply that Hfq can mediate RNA-RNA interactions via base stacking between A-rich RNA sequences. To test if this interaction can occur with physiological sRNAs and target mRNAs, we selected the prominent sRNA-mRNA pair, OxyS and *fhlA*. The *fhlA* mRNA encodes a transcriptional activator of formate metabolism^64^ that is controlled by the central oxidative stress response regulator OxyS. Both OxyS and *fhlA* contain A-rich (ARN)_X_ motifs that are essential for Hfq-binding and RNA pairing *in vivo* ^42, 65^. Curiously, OxyS and *fhlA* share little sequence complementarity; two short (7–9nt long) complementary seed regions can be found at the tips of stable stem-loop structures in both RNAs that were proposed to interact via a “kissing complex”^66^, but the mechanism of OxyS-*fhlA* pairing remains incompletely understood.

**Figure 3:**
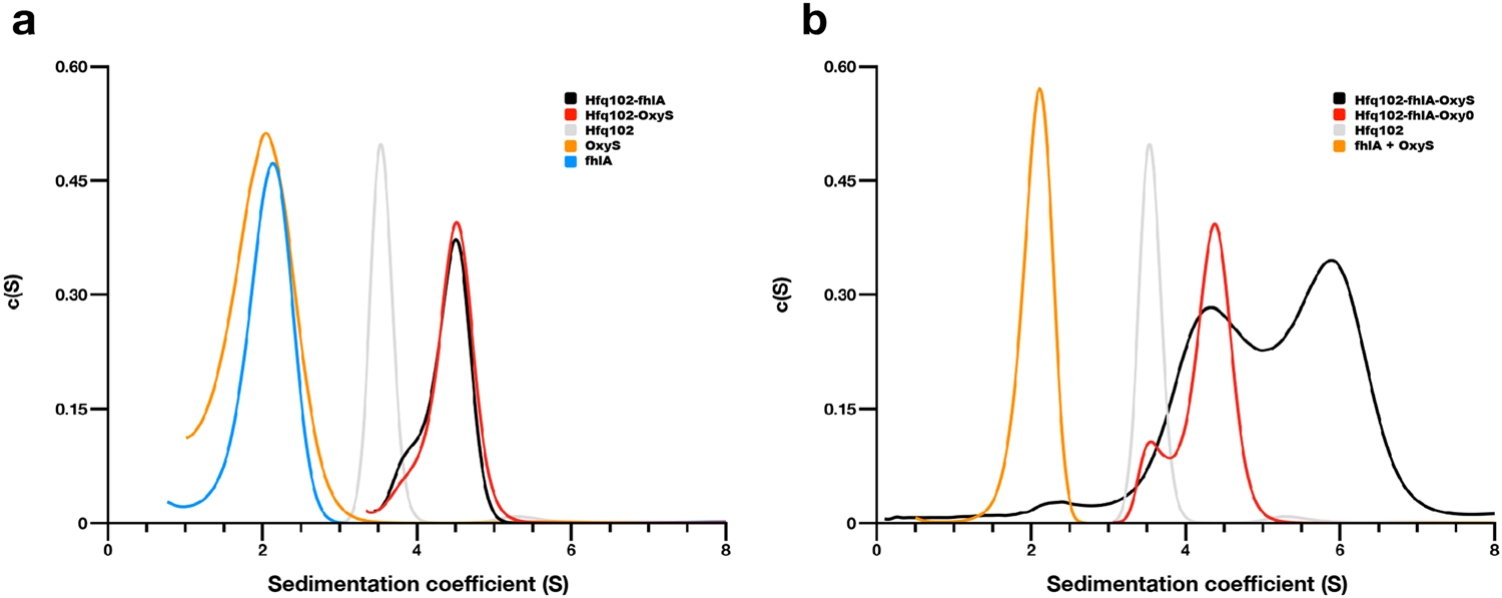
OxyS and *fhlA* can interact via their (ARN)_X_ motifs. AUC curves of Hfq102 complexes with the (ARN)_X_ motifs of OxyS and *fhlA*. All individual species sediment according to their expected sedimentation coefficients and binary complexes sediment as single monomeric Hfq102_6_:RNA species (**a**), but the ternary complex of Hfq102-*fhlA*-OxyS forms a higher order assembly (black in **b**) likely corresponding to Hfq-RNA dimers. This peak is absent with the Oxy0 variant that lacks the N-site bases.

We synthesized oligonucleotides containing the (ARN)_X_ motifs from OxyS (positions 57–86) and *fhlA* (the complementary seed regions were excluded to circumvent interaction by base pairing; see Methods for details). To test the importance of the flipped-out N-site bases, we also created an OxyS variant, Oxy0 where the predicted N-site nucleotides were replaced with abasic linkages (as for ‘AA0’ above). Since the sequence of the OxyS (ARN)_X_ motif is complex and its exact binding mode on Hfq is difficult to predict from the available crystal structures with short OxyS fragments^46^, we manually inspected ARN triplets in the sequence to identify the N-site bases. We focused on the previously annotated ARN region^42^, searched for two purine bases followed by a variable nucleotide and removed the base at this putative N position. The oligonucleotides were differentially labelled with fluorescent probes (Cy5, Cy3), complexed with full length Hfq (Hfq102) alone or in combinations, and their oligomeric states were analysed by AUC (Fig. 3 and Table S1). As expected, Hfq102 alone sedimented as a single hexamer (s = 3.5S) and all RNA molecules revealed a monomeric state (s = 2.0–2.1S). When the (ARN)_X_ segments of OxyS and *fhlA* were mixed without Hfq, they also sedimented as separate monomeric species (s = 2.1S) and did not pair. Remarkably, the Hfq102-*fhlA* and Hfq102-OxyS complexes also revealed simple monomeric Hfq102_6_:RNA complex species (s = 4.5S for both) and did not self-dimerize. This was surprising because Hfq72_6_:A_20_ complexes readily dimerized by themselves in our previous experiments. In contrast, an additional faster sedimenting peak appeared for the ternary Hfq102-*fhlA*-OxyS complex (s = 5.9S), indicating the formation of larger molecular assemblies. Importantly, the Hfq102-*fhlA*-Oxy0 complex did not dimerize (s = 4.4S), again highlighting the importance of the N-site bases.

These results indicate that the Hfq-mediated interactions between the N-site bases of (ARN)_X_ motifs seen in our crystal structure can also occur in OxyS and *fhlA*.

### Conserved (ARN)_X_ motifs are present in a number of sRNAs

The observation of an unanticipated interaction between A-rich sequences in our crystal structure and in solution, prompted us to further explore (ARN)_X_ motifs in Hfq-regulated sRNAs and mRNAs. An increasing body of evidence already recognizes the importance of these motifs for riboregulation in bacteria. For example, it was demonstrated that Hfq binding is specifically enriched at (ARN)_X_ motifs in the 5′ UTR of mRNAs *in vivo* ^34, 37^ and these motifs are essential for their regulation^42, 65, 67, 68^. Several sRNAs were also shown to bind Hfq at (ARN)_X_ motifs that contribute to their stability^21, 40–43^. To explore (ARN)_X_ motifs in sRNAs more broadly, we screened 67 experimentally confirmed sRNAs from *E*. *coli* ^69^. Since existing annotations of (ARN)_X_ containing regions were incomplete and the constraints defining an (ARN)_X_ motif were not clear, we constructed an iterative bioinformatics pipeline that consists of explorative pattern searches with various pattern definitions, secondary structure inspection, and conservation analysis (Figure S5). Based on this, our final pattern described the (ARN)_X_ motif as the concomitant presence of at least 4 ARN triplets within a sequence window of 20 nucleotides, also allowing maximally 2 non-adjacent non-functional triplets and separated single gaps. This pattern is consistent with previous findings that stable RNA binding at Hfq’s distal site involves at least four ARN triplets^30, 35^. All 67 sRNAs were screened with this search pattern independently of whether they are known to interact with Hfq, and the results are summarized in Table 2. From the 67 sRNAs, we have identified matches to the (ARN)_X_ motifs in 25 instances.

**Table 2:** (ARN)_X_ motifs found in *E*. *coli* sRNAs.

Number of identified regions with an (ARN)_X_ motif^a^	sRNA^b^
0	6S, ArcZ, CyaR, DicF, DsrA, FnrS, GadY, GlmY, ISO92, IstR-1, IstR-2, MicA, MicF, OmrA, OmrB, RdlA, RdlB, RdlC, RdlD, RprA, RseX, RybB, RydB, RydC, RyeB, RyhB, RyjB, SgrS, SibC, SibD, SokB, SokC, SokE, SokX, Spot42, SraF, SraG, SroA, SroE, SymR, tff, tp2
1	4.5S (56–69), GcvB (9–24), GlmZ (187–198), MgrR (37–53), MicC (66–93), MicM (26–44), OhsC (21–44), OxyS (59–86), RyjA (84–117), SibA (53–69), SibB (32–51), SibE (43–71), SraB (89–122), SroC (4–19), SroD (31–54), SroG (74–91), SroH (97–126)
2	CsrB (226–240, 294–321), RNaseP (129–144, 245–260), RyeG (71–84, 137–155), RyfA (200–219, 259–278), RydF (50–65, 84–99)
3	—
4	CsrC (17–72, 97–125, 143–156, 202–215),RyeA (1–17, 32–53, 188–204, 217–234), tmRNA (69–95, 174–196, 225–247, 309–328)
5 or more	—

^a^One (ARN)_X_ motif contains at least 4 ARN triplets within a sequence window of 20 nucleotides.

^b^The coordinates of (ARN)_X_ motifs are shown in brackets. Note that overlapping regions of ARN pattern matches were merged together into one single (ARN)_X_ motif.

**Figure 4:**
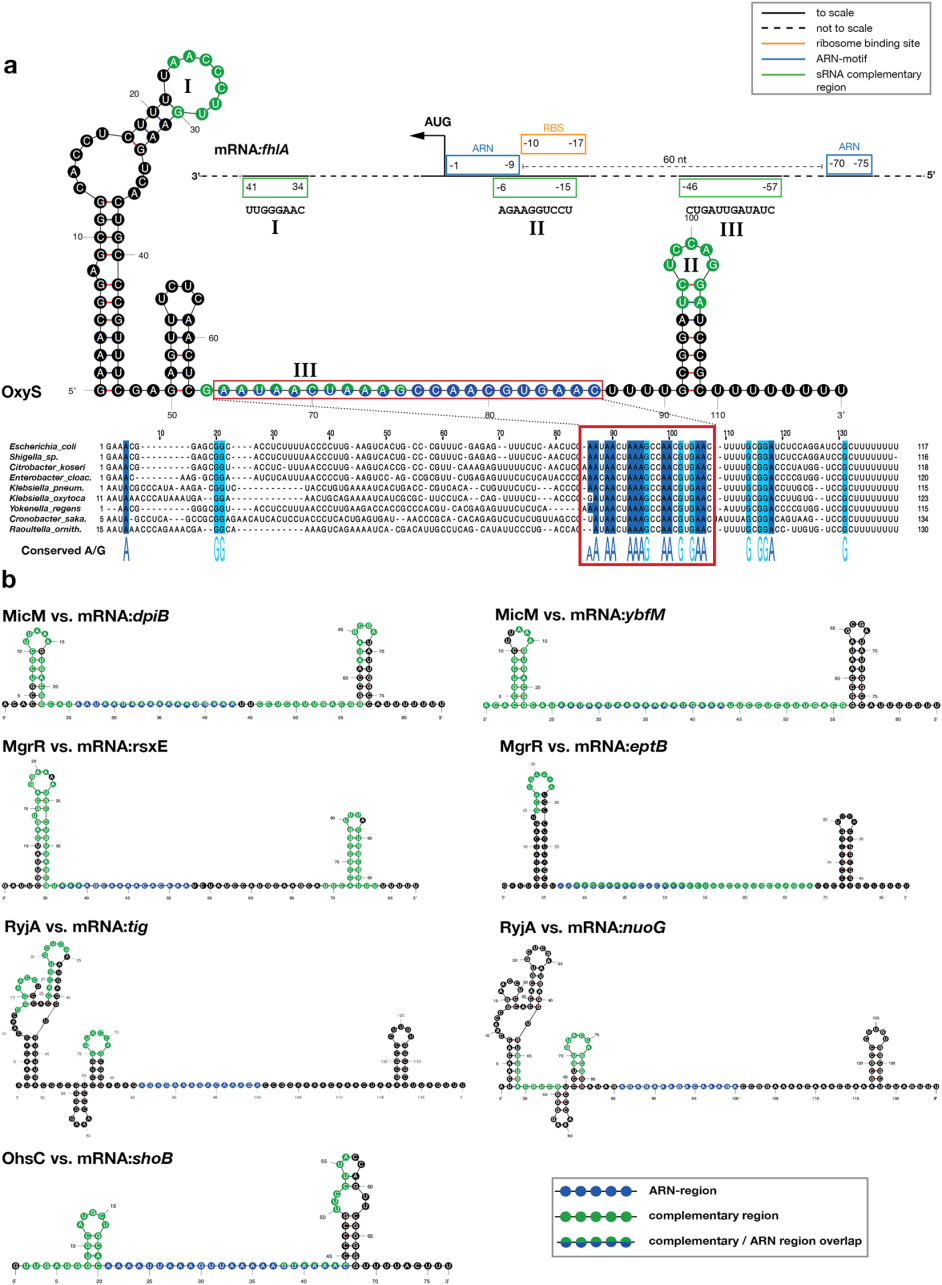
Several (ARN)_X_ motif containing sRNAs share a common architecture. (**a**) Predicted secondary structure of OxyS showing the (ARN)_X_ motif (blue dots) located between two stem loops. Alignment (below) of OxyS sequences from Gram-negative bacteria (*Escherichia coli*, NZ_KE702487.1; *Shigella sp*., NZ_GG657394.1; *Citrobacter koseri*, NC_009792.1; *Enterobacter cloacae*, NC_014618.1; *Klebsiella pneumoniae*, NZ_KI535689.1; *Klebsiella oxytoca*, NZ_JH603150.1; *Yokenella regensburgei*, NZ_JH417870.1; *Cronobacter sakazakii*, NC_020260.1; *Raoultella ornithinolytica*, NC_021066.1) reveals a strong conservation of the (ARN)_X_ motif among various bacteria. Conserved A/G nucleotides are highlighted in blue and marked under the alignment. Seed regions for *fhlA* pairing map to the tips of the stem loops (green dots). The topology of *fhlA* is shown above in 3′ to 5′ orientation. (ARN)_X_ regions (blue boxes) are near the start codon and further upstream in the 5′ UTR. Seed regions (green boxes) are early in the CDS and in the 5′ UTR. Roman numbers (consistent with Table S3) indicate the corresponding complementary sequences between OxyS and *fhlA*. (**b**) Predicted secondary structure of four sRNAs showing strikingly similar architecture to OxyS. See also Figures S6–S10 for more detailed information.

The matching sRNAs include many known Hfq interactors and several previously documented examples of (ARN)_X_ motif-containing sRNAs, such as OxyS^42^ and MicM (also known as ChiX)^40^, as well as several additional instances, where the role of (ARN)_X_ motifs has not yet been implicated. In some sRNAs, we found multiple (two or four) non-adjacent (ARN)_X_ motifs. Interestingly, most identified (ARN)_X_ motifs contained at least 5 ARN triplets, even though our pattern searches required only 4 triplets. Analysing sequence conservation within the ARN triplets, also revealed a preference for A in the R position (Figs 4 and S6–S9, and data not shown), consistent with previous structural^35^ and tryptophan fluorescence quenching data^26^. Of note, we did not find (ARN)_X_ motifs in 42 out of the 67 sRNAs tested, which include several well-studied sRNAs (e.g. RybB, DsrA, and RydC)^27, 30, 55^ that were shown to bind to the proximal and rim sites of Hfq and anneal with mRNAs bound to the distal site of the same Hfq hexamer^21^. The presence of conserved (ARN)_X_ motifs in a distinct subset of sRNAs suggests that these sequence elements may have specific roles in the function of these sRNAs and would merit further investigation.

### Several (ARN)_x_ motif-containing sRNAs share common structural features

To further explore the role of (ARN)_X_ motifs in the above identified set of (ARN)_X_ motif-containing sRNAs, we analysed their secondary structure and the arrangement of known functional modules (e.g. mRNA complementary seed regions) within their sequences. From the 25 sRNAs with predicted (ARN)_X_ motifs, we selected 17 that - for simplicity - contain one single (ARN)_X_ motif (Table 2). With these, we performed secondary structure predictions using three independent thermodynamic folding simulations and mapped the position of the (ARN)_X_ motif relative to secondary structure elements. This showed that the (ARN)_X_ motifs are often flanked by predicted secondary structure elements such as stem loops on one side or both.

Interestingly, we also found that four of the analysed sRNAs (MicM, MgrR, RyjA and OhsC) closely resemble OxyS in their overall structure (Fig. 4). They all feature two stem loops tightly embracing the (ARN)_X_ motif in a spatial arrangement that is so conserved that the different sRNA folds can be directly superimposed. To analyse these five examples (including OxyS) further, we prepared multiple sequence alignments for the sRNAs from related bacteria, which revealed high conservation of the (ARN)_X_ motifs, further supporting their functional importance (Figs 4 and S6–S9). In three out of the five sRNAs, the (ARN)_X_ motif also overlapped with experimentally determined Hfq binding sites (J. Vogel, personal communication)^23, 42^. Next, we mapped functionally relevant sequence regions on these five selected sRNAs. We found that the (ARN)_X_ motif is positioned 20–40 nts away from the 3′ poly(U) tail in all cases (Table S2). This distance appears sufficient to reach between the distal and proximal faces of Hfq, likely allowing the (ARN)_X_ motif and the U-rich tail to bind simultaneously to Hfq. Using complementary search algorithms, we also identified the regions in the five selected sRNAs that are complementary to their well-known target mRNAs (Table S3). We searched with nine mRNAs: *fhlA*, *rpoS*, *shoB*, *ybfM*, *dpiB*, *eptB*, *rsxE*, *tig*, and *nuoG* and mapped the complementary regions onto the sRNA structure. For eight out of the nine sRNA-mRNA pairs (with the exception of the OxyS-*rpoS* pair), complementary regions localized to stem loops flanking the two sides of the (ARN)_X_ motif (Figs 4 and S6–S9).

Taken together, these analyses reveal that unrelated (ARN)_X_ motif-containing sRNAs share a common functional architecture, with a conserved localization of (ARN)_X_ motifs and seed regions within an overall similar structural arrangement.

### mRNA targets of (ARN)_X_ motif-containing sRNAs display common architectural features

*fhlA* was previously shown to have a modular architecture, where several short seed regions flank a bipartite (ARN)_X_ motif involved in Hfq binding^65, 66^. Based on our observation that several (ARN)_X_ motif-containing sRNAs share a common architecture, we wondered if the mRNA targets of these RNAs also share a similar architecture. To check this, we visually located (ARN)_X_ motif containing regions in the respective target mRNAs (Table S4) and mapped these against sRNA complementary regions (Table S3), the ribosome-binding site (RBS), the start codon, and secondary structure elements. The resulting general pattern appears to be more complicated than for (ARN)_X_ motif-containing sRNAs, but a common topology of functional elements can still be observed in eight out of the nine analysed mRNAs (again excluding *rpoS*). In contrast to the one complete (ARN)_X_ motif in (ARN)_X_ motif-containing sRNAs, generally two shorter (ARN)_X_ regions were found in the 5′ UTRs of the target mRNAs (Table S4 and Figure S10). As observed before, one (ARN)_X_ region was typically found close to the start codon and the RBS, while the other is located further upstream (−50 to −140)^23, 37^. In several cases (*fhlA*, *eptB*, and *ybfM*), the predicted (ARN)_X_ regions also overlapped with experimentally identified Hfq-binding sites^23, 65^. The spacing between the two (ARN)_X_ regions was ~60 nts in all cases and often contained stem loops or other folded elements, suggesting that these regions may constitute two parts of a bipartite (ARN)_X_ motif, which could come together in space upon folding of the mRNA (data not shown)^65^. In addition, common features extended to sRNA complementary regions: multiple short seed regions were found in the proximity of (ARN)_X_ motifs, either upstream of the first (ARN)_X_ region, between the two (ARN)_X_ regions, or downstream of the second (ARN)_X_ region at the beginning of the coding sequence (Figure S10). In some cases, seed regions were found overlapping with (ARN)_X_ regions (also observed by Tree *et al*.^37^). Of note, *rpoS* was a clear outlier in our analysis: it contains a long complementary region with OxyS, an (ARN)_X_ region far upstream in the 5′UTR, and a quite different secondary structure (data not shown). However, we observed marked structural similarities in the other mRNA targets of our selected (ARN)_X_ motif-containing sRNAs (Figure S10).

## Discussion

An increasing body of evidence indicates the functional importance of (ARN)_X_ sequence motifs in Hfq-dependent riboregulation in bacteria *in vivo*. These motifs are widespread in Hfq-regulated RNAs in general; they are particularly abundant in the 5′UTRs of mRNAs and are also present in several sRNAs^21, 34, 37, 40, 42^. Previous research has shown that (ARN)_X_ motifs provide essential Hfq binding sites and interact with Hfq’s distal site^31, 42, 65, 67, 68, 70, 71^. Hfq binding involves up to six ARN triplets and occurs on a circular fashion^36^, as seen in the crystal structures (Fig. 1 and Link *et al*.^35^). Interestingly, every third base at the N-site is excluded from Hfq binding and points towards the solvent. In this study, we present a crystal structure of an *E*. *coli* Hfq-A_18_ RNA complex, which reveals an additional structural feature of (ARN)_X_ motifs. It shows that, when bound to Hfq, these motifs can create base-stacking interactions between two RNA molecules (Fig. 1). Surprisingly, the observed interaction is mediated by the flipped-out N-site bases, proposing a functional role for these so far enigmatic residues and their unusual positioning on Hfq’s surface. Compared to previously reported Hfq-poly(A) RNA structures, the orientations of the N-site bases are practically unchanged, suggesting that stacking interactions can be formed without requiring any significant conformational changes after Hfq binding. Remarkably, rotation of the flipped-out base is restricted by the proximity of the protein surface to only a few tens of degrees, suggesting that Hfq actively prepares the observed RNA configuration.

Using abasic RNA probes that specifically lack the N-site bases, we provide several lines of biophysical evidence that support the occurrence of the structurally observed supramolecular interaction in solution and confirm its dependence on the N-site bases. Although in EMSA, ITC and FA experiments the exact composition of the higher order complexes could not be directly determined and the formation of e.g. 2:1 Hfq_6_:RNA complexes that have been observed previously by others^38, 43^ could not be excluded, our AUC experiments strongly suggest a 2:2 complex. 2:1 Hfq_6_:RNA complexes are also thought to have low abundance and little relevance at physiological Hfq-RNA ratios^30, 39, 43, 72^. Furthermore, we show that the assemblies strongly depend on the presence of flipped-out N-site bases in the RNA and their stability scales with the base stacking affinity of these bases. This agrees well with the base-stacking mediated 2:2 assembly in our crystal structure, but is difficult to recapitulate with 2:1 Hfq_6_:RNA complexes as absence of the N-site bases would not be expected to influence tandem binding of two Hfq hexamers on one RNA (binding affinity is not affected; Figures S3 and S4). Finally, our results with the Hfq-*fhlA*-OxyS complex can only be explained with a 2:2 Hfq_6_:RNA assembly (i.e. 2 Hfq_6_: 1 *fhlA*: 1 OxyS), as neither of the two RNAs formed higher order complexes when binding to Hfq individually.

**Figure 5:**
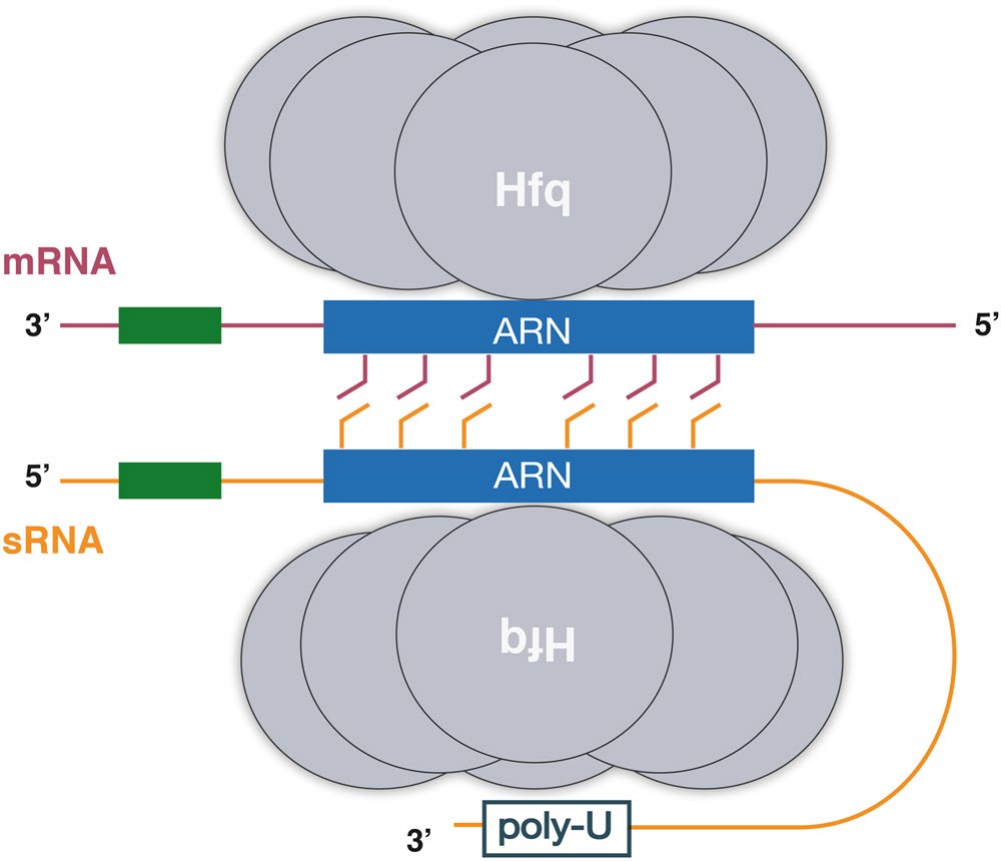
Schematic model for the putative role of (ARN)_X_ motifs in Hfq mediated sRNA-mRNA pairing. Hfq (grey) binding to (ARN)_X_ motifs (blue) in both sRNAs (orange) and mRNAs (dark red) exposes the N-site bases, enabling base stacking between the two RNAs. This (ARN)_X_-mediated interaction can help to initiate first contact between the two RNA molecules, thereby promoting interaction between complementary regions (green boxes) for sRNA-mRNA annealing. Head-to-head arrangement of the Hfq rings guarantees an antiparallel arrangement of the two interacting RNA molecules.

Our bioinformatical analysis of a large set of *E*. *coli* sRNAs revealed that (ARN)_X_ motifs are present in many sRNAs, where they are highly conserved and in some cases co-occur with a specific arrangement of characteristic sequence and secondary structure elements. These observations indicate that (ARN)_X_ motifs can play a role not only in mRNAs, but also in some (ARN)_X_ motif-containing sRNAs. Based on our structural data, we hypothesise that (ARN)_X_ motif-containing sRNAs may bind to Hfq’s distal site and interact with mRNAs that are bound to a separate Hfq hexamer using interlocking base stacking of the flipped-out N-site bases as seen in our crystal structure (Figs 1 and 5). Such interaction between preformed Hfq-RNA complexes may enable association between diverse RNA molecules, allowing them to quickly probe their complementarity; and, in case of a positive match, trigger further annealing of upstream and downstream segments of the affected (ARN)_X_ motif-containing sRNA-mRNA (Fig. 5). If true, this mechanism can provide a platform for rapid partner search on a ‘speed-dating’ fashion.

Of note, the interaction observed in our crystal structure is well suited to initiate RNA-RNA interactions transiently as (i) it occurs between appropriately pre-organized protein-RNA assemblies, (ii) it has low sequence specificity and can bring together a variety of RNAs, (iii) it positions the two RNA molecules in antiparallel orientation, as required for proper pairing^62^, (iv) it has only micro-molar affinity enabling a rapid turnover^73^, (v) it requires additional sequence-specific interactions to create a stable pair for a proper gene regulatory response. This putative mechanism may act in concert with other known annealing pathways, supporting or specifying the function of specific (ARN)_X_ motif containing sRNA. Due to its specific physicochemical properties, ARN base stacking can be particularly beneficial for sRNAs that act on multiple target mRNAs, with most of which they share only little sequence complementarity. Here, base stacking can enable interaction with many potential target RNAs and allow them to find even short complementary matches. In addition, the (ARN)_X_ interactions can also help increase the affinity of these multifaceted sRNAs towards one or another of their targets and thus contribute to their specificity. Consistent with this idea, we find several multi-target sRNA in our list of (ARN)_X_ motif-containing sRNAs (Table 2).

One example of a (ARN)_X_ motif-containing sRNA-mRNA pair is the central oxidative stress response regulator OxyS and its prominent target *fhlA*. Various studies on OxyS-*fhlA* suggested a so-called ‘kissing loop’ annealing model, where short seed regions in stem loops flanking the Hfq binding sites interact with complementary segments in the partner RNA^65, 66^. Now, our structural and biochemical results indicate that Hfq-bound OxyS and *fhlA* can interact via their (ARN)_X_ motifs, perhaps initiating and/or facilitating the full RNA pairing. These results are consistent with previous studies showing that both OxyS and *fhlA* interact with the distal site of Hfq^21, 42, 65^ and can help elaborate their non-canonical mechanism of pairing.

Interestingly, our bioinformatics analysis also revealed that several other (ARN)_X_-containing sRNA-mRNA pairs contain similar architectural features including stem loops and seed sequences flanking the (ARN)_X_ motifs. It will be interesting to test if these pairs can also associate via their (ARN)_X_ motifs and follow annealing mechanisms that are similar to the OxyS-*fhlA* pair.

Our results are consistent with the work of others showing that (ARN)_X_ motifs contribute to Hfq binding and riboregulation in several sRNAs (e.g. OxyS, MgrR, and MicM/ChiX)^21, 42, 68, 74, 75^, whereas these motifs are absent and counterproductive to function if introduced in others (e.g. RyhB, DsrA, RydC, etc.)^21, 27^. In fact, a recent study by Schu *et al*.^21^ showed that the stability and function of a number of sRNAs is compromised in Hfq distal face mutants and suggested that (ARN)_X_ motifs define the stability, target choice and functional role of a specific class of sRNAs (defined as Class II). In agreement with these studies, we find (ARN)_X_ motif matches in prominent Class II sRNAs (such as MicM and MgrR). Furthermore, Schu et al. also proposed that Class II sRNAs interact with mRNA targets bound to the rim of Hfq, in contrast to sRNAs without (ARN)_X_ motifs (Class I) that bind to the proximal and rim sites and anneal with mRNA targets bound to Hfq’s distal site. As the rim site of Hfq is smaller and weaker than the other binding sites and many Class II mRNA targets also have (ARN)_X_ motifs^21^, the ARN-ARN interactions observed here may help initiate or stabilize sRNA-mRNA contacts, thereby contributing to Class II sRNA function. In addition, several sRNAs, including OxyS, showed intermediate behaviours in the studies of Schu *et al*.^21^, suggesting that the mechanistic diversity of sRNA-mRNA pairing may be even greater. Our ARN pattern search protocol can help identify (ARN)_X_ motifs in bacterial RNAs more broadly, thus helping to classify sRNAs and derive testable hypotheses for their functional and mechanistic features. In accord, our ARN-containing RNA set also contains sRNAs that have not yet been implicated to interact with Hfq, and it will be interesting to test if these RNAs may rely on Hfq under specific cellular conditions.

We speculate that the proposed RNA interaction model may be relevant in different Gram - negative bacteria, as the binding mode of poly(A) RNA is shared and our bioinformatics analysis revealed conservation of (ARN)_X_ motifs in these species. Hfq proteins in Gram - positive bacteria bind RNA differently at their distal site, relying on a bipartite RNA-binding motif with no flipped-out bases; thus, our model is probably not applicable to these species. However, to investigate the exact impact of Hfq-mediated ARN base stacking *in vivo* and its species-specific features, further studies will be required.

One of our most surprising results is that Hfq can use base stacking to mediate RNA-RNA interactions. Base stacking is prominent in DNA, where it provides a major force stabilizing the structure of the double helix. In RNA, it was observed in structured tRNAs, rRNAs, ribozymes and in the ribosome^76–78^ and has accessory roles in organizing the tertiary fold. Now, we show that base stacking is not a sole property of complex folded RNAs, but it can also occur between two separate single-stranded RNAs if supported by the RNA-chaperone protein Hfq.

The putative role of N-site base stacking in sRNA-mRNA interactions naturally raises the question if the identity of these bases matters for sRNA-mRNA pairing. In other words, are all bases and base combinations at the N-site able to interact equally well or does the identity of these bases convey a hidden code? If such a hidden code exists, this could contribute to specificity of sRNA-mRNA pairing and help ensure the selectivity of gene regulation. Our observations that poly(A) and ‘AAG’ containing RNAs interact more strongly than ‘AAC’ or ‘AAU’ sequences support this hidden code idea. Such preference for the pyrimidine bases can be explained by their advantageous base stacking and hydrogen bonding properties that help keep the RNA-mediated dimeric complex together. Consistently, we find that neither Hfq-OxyS nor Hfq-*fhlA* complexes can self-pair, only their ternary complex forms base stacked Hfq_6_:RNA dimers. However, elucidating the exact role of base stacking in sRNA-mRNA pairing and the principles of their specificity will require substantial further analysis.

## Materials and Methods

### RNA oligonucleotides

All RNA oligonucleotides were synthesized by Integrated DNA Technologies (IDT; Leuven –Belgium). A_30_ and A_20_ contain 30 and 20 consecutive adenine nucleotides, respectively. The ‘AA0’ RNA had the following sequence: (AA0)_6_AA, where 0 denotes an abasic nucleotide. Similarly, ‘AAC’, ‘AAU’ and ‘AAG’ sequences were (AAC)_6_AA, (AAU)_6_AA and (AAG)_6_AA, respectively. The oligonucleotide representing the ARN motifs of OxyS was derived from Gottesman *et al*.^64^ and comprises nucleotides 57 to 86 of full length OxyS, giving rise to the sequence: 5′-UCAACUCGAAUAACUAAAGCCAACGUGAAC-3′. In Oxy0, presumed N-site bases were exchanged for abasic nucleotides, denoted by 0: 5′-UCAA0UC0AA0AA0UAA0GCCAA0GU0AA-3′. The *fhlA* ARN segment oligonucleotide was constructed based on Salim *et al*.^65^ and comprises the two (ARN)_X_ regions (nucleotides −78 to −65 and −14 to +5) directly fused to each other, giving rise to the sequence: 5′-CUAAUAAAAUUCUACCUAGAAGAACAAAAUGUC-3′. Residues −64 to −13 were replaced by a CC-dinucleotide, G at position −11 was replaced for A. For analytical ultracentrifugation, OxyS, Oxy0 and *fhlA* were synthesized with Cy5- and Cy3 fluorescence labels at their 3′-end, respectively. A modified A_20_ RNA, with an ATTO488-dye at the 3′-end was used for fluorescence anisotropy measurements.

### Protein production and crystallization

DNA encoding *Escherichia coli* Hfq72 (containing amino acids 1–72), Hfq102 (full length, aa 1–102), Hfq102^R16A,R17A^ (full length, R16A-R17A solubility mutant), and poly(A)-polymerase 1 (PAP-1; aa 19–478) were cloned into pETM28-SUMO vector and the 6xHis-SUMO-tagged proteins were expressed in *E*. *coli* BL21(DE3) cells in TB medium at 37 °C for 20 h (all three Hfq constructs) or 4 h (the PAP-1 construct). The cell lysate was applied to Ni-Sepharose column (His-Trap, GE Healthcare) in 0.1 M Hepes/NaOH pH 8.0, 0.5 M NaCl, 0.005 M TCEP. To remove nucleic acid contamination, the proteins were washed with 1 M LiCl on the column before eluting with imidazole. The eluate was then incubated with SenP2 protease (1:100) for 18 h at 4 °C and the cleaved SUMO-tag was removed via a second Ni purification. Proteins were further purified by size exclusion chromatography on a Superdex 200 column (0.05 M Hepes/NaOH pH 8.0, 0.5 M NaCl), concentrated to 10 mg/ml, and stored in 0.05 M Hepes/NaOH pH 8.0, 0.5 M NaCl at −80 °C until further use. For poly(A)-polymerase 1, a Heparin-Sepharose purification was included (using 0.05 M Hepes/NaOH pH 8.0, 0.5 M NaCl–2 M NaCl) after SenP2 cleavage to better remove the cleaved SUMO-tag and nucleic acid contaminations.

For crystallization, complexes were formed by mixing Hfq72, A_30_ RNA and PAP-1 in a 1:1.2:1 molar ratio in HS-buffer (2 M NaCl, 0.02 M Hepes pH 8.0, 0.005 M MgCl_2_, 5% Glycerol) and dialyzing the solution against CX-buffer (0.25 M NaCl, 0.02 M Hepes pH 8.0, 0.05 M MgCl_2_, 10% Glycerol), and concentrated to 5 mg/ml. Crystals were grown at 20 °C in hanging drop vapor diffusion plates combining equal volumes of the complex solution with the well solution containing 0.1 M phosphate-citrate buffer pH 4.2, 27% PEG 1000, and 0.2 M LiSO_4_.

### Data collection and structure determination

Crystals were cryo-protected with 12% 2,3-butanediol in the well solution and flash frozen in liquid nitrogen. X-ray data collection was performed at 100 K; diffraction images were collected at BM30A (ESRF, Grenoble). Diffraction data was processed to 2.5 Å resolution with XDS^79^. Even though the signal to noise ratio was still quite high at this resolution, the data was cut due to low completeness in the high resolution range (Table 1). The latter was probably caused by suboptimal placement of the X-ray detector during data collection, precluding collection of all diffraction data to the highest possible resolution. The structure was solved by molecular replacement in PHASER using the unliganded *E*. *coli* Hfq structure as a search model (PDB-ID:1HK9)^80, 81^. The crystals belonged to space group R32 and the asymmetric unit contained two Hfq72 subunits bound to two adenine oligonucleotides, with crystallographic symmetry generating the biologically relevant homo-hexamers and the complete A_18_ chain. Model building in COOT^82^ was alternated with refinement in PHENIX^83^ until the R-values converged (Table 1). The structure was validated with MOLPROBITY^84^. Structure factors and coordinates have been deposited with the Protein Data Bank under accession number 5NEW. Molecular images were generated in PyMOL^85^.

### Size exclusion chromatography (SEC)

Hfq72-RNA complexes were prepared at 10 µM concentration in AUC-buffer (0.25 M NaCl, 0.02 M Hepes/NaOH pH 8.0, 0.005 M MgCl_2_, 2% Glycerol) and run at 0.05 ml/min on a Superdex S200 10/300 (GE Healthcare) pre-equilibrated in AUC buffer. UV-absorbance data were collected at 280 nm and 254 nm respectively.

### Analytical ultracentrifugation (AUC)

Hfq72-RNA and Hfq102-RNA complexes were prepared in AUC-buffer (0.25 M NaCl, 0.02 M Hepes pH 8.0, 0.005 M MgCl_2_, 2% Glycerol) at a concentration of 33 µM. Sedimentation velocity experiments were performed at 20 °C in a Beckman OptimaXL-A centrifuge fitted with a four-hole AN-60 rotor and double-sector Epon centerpieces at 45 000 rpm. To unambiguously assign the composition of the complexes, absorbance data were collected at 280 nm for RNA-free Hfq, at 254 nm for samples containing unlabelled RNA or at 548 nm or 650 nm for fluorescent oligos. Data were analysed by the c(s) method using the Sedfit software package^86^. The observed s-values were compared with theoretical sedimentation coefficients calculated from our Hfq72-A_20_ crystal structure using HYDROPRO 5a^87, 88^. The viscosity (1.087 mPa·s) and the density (1.015 g/ml) of the AUC-buffer were calculated using the program SEDNTERP V1.09 (J.Philo, D. Hayes, T. Laue). The partial specific volumes were 0.530 ml/g for the RNA, 0.747 ml/g for the protein and 0.721 ml/g for the complex.

### Electrophoretic mobility shift assays (EMSA)

Previous EMSA experiments indicated that full length Hfq102 migrates more effectively into native polyacrylamide gels than the truncated Hfq72 variant (see e.g. Updegrove *et al*.^39^). It was also previously shown that mutation of two arginine residues, R16 and R17 to alanine in *E*. *coli* Hfq reduces non-specific protein aggregation. Therefore, to ensure that potential higher order assemblies do not result from protein aggregation in electrophoretic mobility shift assays, we used a Hfq102^R16A,R17A^ mutant^30, 61, 62^. These mutations at the Hfq’s rim site do not affect binding of A-rich RNA^21^. Hfq102^R16A,R17A^ was expressed and purified as described above. ssRNA substrates were 5′-^32^P-labelled using T4 Polynucleotide Kinase (NEB) and [γ-^32^P]-ATP (Hartmann Analytic), and then purified on a Bio-Spin 6 column (Bio-Rad) following the manufacturers recommendations. Radiolabelled RNA was then incubated with varying amounts of Hfq102^R16A,R17A^ in EMSA-buffer (0.25 M NaCl, 0.05 M Tris pH 7.5, 10% (v/v) Glycerol) for 30 min at 25 °C. Each 10 µl reaction contained 1 µl of 200 nM labelled ssRNA and an increasing excess of Hfq102^R16A,R17A^ (1 μl of 20 nM–2000 nM). Complexes were separated via polyacrylamide gel electrophoresis using native 4–20% gradient gels in 1xTBE running buffer and results were imaged on a Typhoon FLA 9500 phosphoimager.

### Isothermal titration calorimetry (ITC)

ITC experiments were carried out with an ITC_200_ microcalorimeter (GE-Healthcare; Microcal) at 25 °C in AUC buffer (0.25 M NaCl, 0.02 M Hepes/NaOH pH 8.0, 0.005 M MgCl_2_, 2% Glycerol) after intensive dialysis of both Hfq72 and RNA overnight. The RNA was loaded in the sample cell at a concentration of 10 µM and was titrated with 150 µM protein solution from the injection syringe. The heat of dilution was measured in control titrations with buffer and subtracted from the binding data. Data were analysed using the Origin 7.0 (Microcal) software. After testing several binding models, the Hfq72-‘AA0’ binding data was best fit by a ‘one-set-of-binding-sites’ model, while the Hfq72-A_20_ ITC data corresponded best to a ‘two-set-of-binding-sites’ model.

### Fluorescence anisotropy measurements

For fluorescence anisotropy, Hfq72 was dialyzed against AUC buffer. Starting from a concentration of 195 µM, Hfq72 was serially diluted by a factor of 0.66 in AUC buffer supplemented with BSA at a final concentration of 1 g/l. The resulting solutions were mixed with 3′-ATTO488-labelled A_20_ (2 nM) in a final volume of 150 µl. Samples were prepared in triplicates in 96 - well plates. Anisotropy measurements were conducted in an Infinite M1000 plate reader (TECAN) at 25 °C. Excitation wavelength was 470 nm and the emitted light was recorded at 530 nm. Data were processed and fit according to a ‘two-set-of sites’-model in the GraphPad Prism software package.

### Creation of permuted ARN pattern sets for sRNA sequence analysis

Since the rules defining an (ARN)_X_ motif were unknown, in order to survey the occurrence of (ARN)_X_ motifs in sRNAs comprehensively we designed and implemented a custom algorithm that created distinct sets of ARN patterns with several different pattern definitions.

Our pattern definitions varied primarily in their degrees of ambiguity. The starting point was a conservative ARN pattern definition containing six consecutive ARN triplets directly following each other. Then, several properties of the pattern were defined in a more permissive manner, in a way that was consistent with known examples of ARN motif sequences. First, one or two non-functional (non-ARN) triplets were allowed within the pattern. Second, one or two single nucleotide gaps were introduced next to ARN triplets. Third, single gaps were allowed anywhere in the pattern. Forth, combinations of the above-described different ambiguity properties were also allowed.

Using these pattern definitions, we then algorithmically created comprehensive pattern sets by permuting the combinations and positions of non-ARN elements (triplets or gaps) within the patterns. The resulting ensemble of pattern sets allowed us to cover the entire possible diversity that may occur in a potential (ARN)_X_ motif and was used for pattern matching in sRNA sequences to produce distinct sets of results for each pattern definition.

### ARN pattern search and bioinformatic analysis

*E*. *coli* sRNA sequences were extracted from the Storz lab resource^69^ and homologous sRNA sequences in other bacteria were identified using BLASTN in NCBI and KEGG^89–91^. We did not attempt to sort the sRNA dataset into positive and negative interactors, as negatives under one condition may interact under different cell or experimental conditions, as was recently seen with McaS^21, 92^. Sequences were aligned with Clustal W 2.0^93^ and displayed in Jalview^94^. *E*. *coli* mRNA sequence data was retrieved from Genolist^95^ and analysed within a sequence window containing the 5′ UTR and the first 80 nts (+1–+80) of the coding sequence. In cases where 5′ UTR annotation was not available (e.g. for downstream genes in multigene operons), position −80 was used as a default starting point. These coordinate ranges were chosen to include all known functional regions of well-annotated mRNAs (such as *fhlA*) and exceed them by a safety margin.

Bioinformatic analysis was conducted in several steps using custom designed Perl algorithms and the overall pipeline is illustrated in Figure S5. For sRNAs, ARN pattern searches were performed in iterative fashion. To generate the initial patterns, we analysed experimentally validated (ARN)_X_ regions as well as the requirements of strong Hfq binding at this region, and algorithmically generated a number of ARN pattern definitions containing different numbers of ARN triplets in combination with non-ARN triplets and distinct single gaps. With these, we performed pattern matching in sRNAs to get an overview of the distribution of different kinds of ARN motifs in these sequences. This survey identified commonly observed patterns, which were then further refined iteratively by analysing similarities in the identified candidate (ARN)_X_ regions, conservation of their sequence, and their structural features. The resulting refined ARN pattern was then used to annotate (ARN)_X_ motifs in 67 sRNAs. Overlapping matches were merged into a single (ARN)_X_ region (Table 2).

Secondary structure predictions were performed using KineFold, Mfold, and RNAfold^96–98^, where (ARN)_X_ motifs were protected from base-pairing. Plots were generated with Mfold and colour-coding was added manually. For identifying complementary regions between sRNAs and their target mRNAs, we defined the seed region in our search algorithm to minimally contain either seven consecutive base pairs or six consecutive base pairs flanked by a single gap followed by two base pairs.

### Accession codes

Coordinates and structure factors have been deposited in the Protein Data Bank under accession code 5NEW.

## Electronic supplementary material

Supplementary Information

## References

[1] Lenz DH (2004). The small RNA chaperone Hfq and multiple small RNAs control quorum sensing in Vibrio harveyi and Vibrio cholerae. Cell.

[2] Shakhnovich EA, Davis BM, Waldor MK (2009). Hfq negatively regulates type III secretion in EHEC and several other pathogens. Molecular microbiology.

[3] Papenfort K, Sun Y, Miyakoshi M, Vanderpool CK, Vogel J (2013). Small RNA-mediated activation of sugar phosphatase mRNA regulates glucose homeostasis. Cell.

[4] Bobrovskyy M, Vanderpool CK (2013). Regulation of bacterial metabolism by small RNAs using diverse mechanisms. Annu Rev Genet.

[5] Altuvia S, Weinstein-Fischer D, Zhang A, Postow L, Storz G (1997). A small, stable RNA induced by oxidative stress: role as a pleiotropic regulator and antimutator. Cell.

[6] Chao Y, Vogel J (2010). The role of Hfq in bacterial pathogens. Current opinion in microbiology.

[7] Storz G, Vogel J, Wassarman KM (2011). Regulation by small RNAs in bacteria: expanding frontiers. Molecular cell.

[8] Wilson JW (2007). Space flight alters bacterial gene expression and virulence and reveals a role for global regulator Hfq. Proceedings of the National Academy of Sciences of the United States of America.

[9] Chambers JR, Sauer K (2013). Small RNAs and their role in biofilm formation. Trends Microbiol.

[10] Jorgensen MG (2012). Small regulatory RNAs control the multi-cellular adhesive lifestyle of Escherichia coli. Mol Microbiol.

[11] Sharma CM, Vogel J (2009). Experimental approaches for the discovery and characterization of regulatory small RNA. Curr Opin Microbiol.

[12] Melamed S (2016). Global Mapping of Small RNA-Target Interactions in Bacteria. Mol Cell.

[13] Han K, Tjaden B, Lory S (2016). GRIL-seq provides a method for identifying direct targets of bacterial small regulatory RNA by *in vivo* proximity ligation. Nat Microbiol.

[14] Papenfort K, Bouvier M, Mika F, Sharma CM, Vogel J (2010). Evidence for an autonomous 5′ target recognition domain in an Hfq-associated small RNA. Proc Natl Acad Sci USA.

[15] Peterman N, Lavi-Itzkovitz A, Levine E (2014). Large-scale mapping of sequence-function relations in small regulatory RNAs reveals plasticity and modularity. Nucleic Acids Res.

[16] Ansong C (2009). Global systems-level analysis of Hfq and SmpB deletion mutants in Salmonella: implications for virulence and global protein translation. PloS one.

[17] Bilusic I, Popitsch N, Rescheneder P, Schroeder R, Lybecker M (2014). Revisiting the coding potential of the E. coli genome through Hfq co-immunoprecipitation. RNA Biol.

[18] Chambers, J. R. & Bender, K. S. The RNA Chaperone Hfq Is Important for Growth and Stress Tolerance in Francisella novicida. *PloS one* **6**, doi:10.1371/journal.pone.0019797 (2011). 10.1371/journal.pone.0019797 PMC3088715 21573133

[19] Vogel J, Luisi BF (2011). Hfq and its constellation of RNA. Nature reviews. Microbiology.

[20] Moller T (2002). Hfq: a bacterial Sm-like protein that mediates RNA-RNA interaction. Molecular cell.

[21] Schu DJ, Zhang A, Gottesman S, Storz G (2015). Alternative Hfq-sRNA interaction modes dictate alternative mRNA recognition. EMBO J.

[22] Weichenrieder O (2014). RNA binding by Hfq and ring-forming (L)Sm proteins: a trade-off between optimal sequence readout and RNA backbone conformation. RNA Biol.

[23] Holmqvist E (2016). Global RNA recognition patterns of post-transcriptional regulators Hfq and CsrA revealed by UV crosslinking *in vivo*. EMBO J.

[24] Updegrove TB, Zhang A, Storz G (2016). Hfq: the flexible RNA matchmaker. Curr Opin Microbiol.

[25] Beich-Frandsen M (2011). Structural insights into the dynamics and function of the C-terminus of the E. coli RNA chaperone Hfq. Nucleic acids research.

[26] Robinson KE, Orans J, Kovach AR, Link TM, Brennan RG (2014). Mapping Hfq-RNA interaction surfaces using tryptophan fluorescence quenching. Nucleic acids research.

[27] Dimastrogiovanni, D. *et al*. Recognition of the small regulatory RNA RydC by the bacterial Hfq protein. *Elife* **3**, doi:10.7554/eLife.05375 (2014). 10.7554/eLife.05375 PMC4337610 25551292

[28] Vecerek B, Rajkowitsch L, Sonnleitner E, Schroeder R, Blasi U (2008). The C-terminal domain of Escherichia coli Hfq is required for regulation. Nucleic Acids Res.

[29] Panja S, Schu DJ, Woodson SA (2013). Conserved arginines on the rim of Hfq catalyze base pair formation and exchange. Nucleic Acids Res.

[30] Sauer, E., Schmidt, S. & Weichenrieder, O. Small RNA binding to the lateral surface of Hfq hexamers and structural rearrangements upon mRNA target recognition. *Proceedings of the National Academy of Sciences of the United States of America*, doi:10.1073/pnas.1202521109 (2012). 10.1073/pnas.1202521109 PMC3386104 22645344

[31] Zhang A, Schu DJ, Tjaden BC, Storz G, Gottesman S (2013). Mutations in interaction surfaces differentially impact E. coli Hfq association with small RNAs and their mRNA targets. J Mol Biol.

[32] Sauer E, Weichenrieder O (2011). Structural basis for RNA 3′-end recognition by Hfq. Proceedings of the National Academy of Sciences of the United States of America.

[33] Otaka H, Ishikawa H, Morita T, Aiba H (2011). PolyU tail of rho-independent terminator of bacterial small RNAs is essential for Hfq action. Proc Natl Acad Sci USA.

[34] Lorenz C (2010). Genomic SELEX for Hfq-binding RNAs identifies genomic aptamers predominantly in antisense transcripts. Nucleic acids research.

[35] Link TM, Valentin-Hansen P, Brennan RG (2009). Structure of Escherichia coli Hfq bound to polyriboadenylate RNA. Proceedings of the National Academy of Sciences of the United States of America.

[36] de Haseth PL, Uhlenbeck OC (1980). Interaction of Escherichia coli host factor protein with oligoriboadenylates. Biochemistry.

[37] Tree JJ, Granneman S, McAteer SP, Tollervey D, Gally DL (2014). Identification of Bacteriophage-Encoded Anti-sRNAs in Pathogenic Escherichia coli. Molecular cell.

[38] Wang W (2011). Cooperation of Escherichia coli Hfq hexamers in DsrA binding. Genes Dev.

[39] Updegrove TB, Correia JJ, Chen Y, Terry C, Wartell RM (2011). The stoichiometry of the Escherichia coli Hfq protein bound to RNA. RNA.

[40] Figueroa-Bossi N, Valentini M, Malleret L, Fiorini F, Bossi L (2009). Caught at its own game: regulatory small RNA inactivated by an inducible transcript mimicking its target. Genes & development.

[41] Moon K, Gottesman S (2011). Competition among Hfq-binding small RNAs in Escherichia coli. Molecular microbiology.

[42] Zhang A, Wassarman KM, Ortega J, Steven AC, Storz G (2002). The Sm-like Hfq protein increases OxyS RNA interaction with target mRNAs. Molecular cell.

[43] Henderson CA (2013). Hfq binding changes the structure of Escherichia coli small noncoding RNAs OxyS and RprA, which are involved in the riboregulation of rpoS. RNA.

[44] Altuvia S, Zhang A, Argaman L, Tiwari A, Storz G (1998). The Escherichia coli OxyS regulatory RNA represses fhlA translation by blocking ribosome binding. The EMBO journal.

[45] Zhang A (1998). The OxyS regulatory RNA represses rpoS translation and binds the Hfq (HF-I) protein. The EMBO journal.

[46] Wang L (2015). Structural insights into the recognition of the internal A-rich linker from OxyS sRNA by Escherichia coli Hfq. Nucleic Acids Res.

[47] Sobrero P, Valverde C (2012). The bacterial protein Hfq: much more than a mere RNA-binding factor. Crit Rev Microbiol.

[48] Fender A, Elf J, Hampel K, Zimmermann B, Wagner EG (2010). RNAs actively cycle on the Sm-like protein Hfq. Genes & development.

[49] Santiago-Frangos A, Kavita K, Schu DJ, Gottesman S, Woodson SA (2016). C-terminal domain of the RNA chaperone Hfq drives sRNA competition and release of target RNA. Proc Natl Acad Sci USA.

[50] Masse E, Escorcia FE, Gottesman S (2003). Coupled degradation of a small regulatory RNA and its mRNA targets in Escherichia coli. Genes & development.

[51] Bandyra KJ, Luisi BF (2013). Licensing and due process in the turnover of bacterial RNA. RNA Biol.

[52] Papenfort K (2006). SigmaE-dependent small RNAs of Salmonella respond to membrane stress by accelerating global omp mRNA decay. Mol Microbiol.

[53] Vanderpool CK, Gottesman S (2004). Involvement of a novel transcriptional activator and small RNA in post-transcriptional regulation of the glucose phosphoenolpyruvate phosphotransferase system. Mol Microbiol.

[54] Fei J (2015). RNA biochemistry. Determination of *in vivo* target search kinetics of regulatory noncoding RNA. Science.

[55] Wang W, Wang L, Wu J, Gong Q, Shi Y (2013). Hfq-bridged ternary complex is important for translation activation of rpoS by DsrA. Nucleic acids research.

[56] Schumacher MA, Pearson RF, Moller T, Valentin-Hansen P, Brennan RG (2002). Structures of the pleiotropic translational regulator Hfq and an Hfq-RNA complex: a bacterial Sm-like protein. The EMBO journal.

[57] Vincent, H. A. *et al*. Characterization of Vibrio cholerae Hfq Provides Novel Insights into the Role of the Hfq C-Terminal Region. *Journal of molecular biology*, doi:10.1016/j.jmb.2012.03.028 (2012). 10.1016/j.jmb.2012.03.028 PMC3477312 22484176

[58] Sun X, Zhulin I, Wartell RM (2002). Predicted structure and phyletic distribution of the RNA-binding protein Hfq. Nucleic acids research.

[59] Rajkowitsch L, Schroeder R (2007). Dissecting RNA chaperone activity. RNA.

[60] Howlett GJ, Minton AP, Rivas G (2006). Analytical ultracentrifugation for the study of protein association and assembly. Curr Opin Chem Biol.

[61] Panja S, Woodson SA (2012). Hexamer to monomer equilibrium of E. coli Hfq in solution and its impact on RNA annealing. Journal of molecular biology.

[62] Panja, S. & Woodson, S. A. Hfq proximity and orientation controls RNA annealing. *Nucleic acids research*, doi:10.1093/nar/gks618 (2012). 10.1093/nar/gks618 PMC3458560 22761405

[63] Friedman RA, Honig B (1995). A free energy analysis of nucleic acid base stacking in aqueous solution. Biophys J.

[64] Gottesman, S. The small RNA regulators of *Escherichia coli*: roles and mechanisms. *Annual review of microbiology* **58**, 303–328, doi:10.1146/annurev.micro.58.030603.123841 (2004). 10.1146/annurev.micro.58.030603.123841 15487940

[65] Salim, N. N. & Feig, A. L. An upstream Hfq binding site in the fhlA mRNA leader region facilitates the OxyS-fhlA interaction. *PloS one* **5**, doi:10.1371/journal.pone.0013028 (2010). 10.1371/journal.pone.0013028 PMC2946933 20927406

[66] Argaman L, Altuvia S (2000). fhlA repression by OxyS RNA: kissing complex formation at two sites results in a stable antisense-target RNA complex. Journal of molecular biology.

[67] Soper TJ, Woodson SA (2008). The rpoS mRNA leader recruits Hfq to facilitate annealing with DsrA sRNA. RNA.

[68] Updegrove T, Wilf N, Sun X, Wartell RM (2008). Effect of Hfq on RprA-rpoS mRNA pairing: Hfq-RNA binding and the influence of the 5′ rpoS mRNA leader region. Biochemistry.

[69] Storz, G. *E*. *coli small RNAs* http://cbmp.nichd.nih.gov/segr/ecoli_rnas.html (2010).

[70] Salim, N. N., Faner, M. A., Philip, J. A. & Feig, A. L. Requirement of upstream Hfq-binding (ARN)x elements in glmS and the Hfq C-terminal region for GlmS upregulation by sRNAs GlmZ and GlmY{dagger}. *Nucleic acids research*, doi:10.1093/nar/gks392 (2012). 10.1093/nar/gks392 PMC3439879 22661574

[71] Beisel CL, Updegrove TB, Janson BJ, Storz G (2012). Multiple factors dictate target selection by Hfq-binding small RNAs. EMBO J.

[72] Ribeiro Ede A (2012). Structural flexibility of RNA as molecular basis for Hfq chaperone function. Nucleic acids research.

[73] Lim, W., Mayer, B. & Pawson, T. *Cell Signaling: principles and mechanisms*. (Garland Science, 2014).

[74] Ellis MJ, Trussler RS, Haniford DB (2015). Hfq binds directly to the ribosome-binding site of IS10 transposase mRNA to inhibit translation. Mol Microbiol.

[75] Malecka EM, Strozecka J, Sobanska D, Olejniczak M (2015). Structure of bacterial regulatory RNAs determines their performance in competition for the chaperone protein Hfq. Biochemistry.

[76] Toor N, Keating KS, Taylor SD, Pyle AM (2008). Crystal structure of a self-spliced group II intron. Science.

[77] Quigley GJ, Rich A (1976). Structural domains of transfer RNA molecules. Science.

[78] Noller HF (2005). RNA structure: reading the ribosome. Science.

[79] Kabsch W (2010). Xds. Acta Crystallographica Section D-Biological Crystallography.

[80] Sauter C, Basquin J, Suck D (2003). Sm-like proteins in Eubacteria: the crystal structure of the Hfq protein from Escherichia coli. Nucleic acids research.

[81] Mccoy AJ (2007). Phaser crystallographic software. Journal of Applied Crystallography.

[82] Emsley P, Lohkamp B, Scott WG, Cowtan K (2010). Features and development of Coot. Acta Crystallographica Section D-Biological Crystallography.

[83] Adams PD (2010). PHENIX: a comprehensive Python-based system for macromolecular structure solution. Acta Crystallogr D Biol Crystallogr.

[84] Chen VB (2010). MolProbity: all-atom structure validation for macromolecular crystallography. Acta Crystallographica Section D-Biological Crystallography.

[85] DeLano, W. L. The PyMol Molecular Viewer. *DeLano Scientific, San Carlos, California*, *USA* www.pymol.org (2002).

[86] Schuck P (2000). Size-distribution analysis of macromolecules by sedimentation velocity ultracentrifugation and lamm equation modeling. Biophysical journal.

[87] Garcia de la Torre, J., Huertas, M. L. & Carrasco, B. Calculation of hydrodynamic properties of globular proteins from their atomic-level structure. *Biophys J* **78**, 719–730, doi:10.1016/S0006-3495(00)76630-6 (2000). 10.1016/S0006-3495(00)76630-6 PMC1300675 10653785

[88] Ortega A, Amoros D, Garcia de la Torre J (2011). Prediction of hydrodynamic and other solution properties of rigid proteins from atomic- and residue-level models. Biophys J.

[89] Kanehisa M (2014). Data, information, knowledge and principle: back to metabolism in KEGG. Nucleic acids research.

[90] Kanehisa M, Goto S (2000). KEGG: kyoto encyclopedia of genes and genomes. Nucleic acids research.

[91] Altschul SF, Gish W, Miller W, Myers EW, Lipman DJ (1990). Basic local alignment search tool. Journal of molecular biology.

[92] Zhang A (2003). Global analysis of small RNA and mRNA targets of Hfq. Mol Microbiol.

[93] Larkin MA (2007). Clustal W and Clustal X version 2.0. Bioinformatics.

[94] Waterhouse AM, Procter JB, Martin DM, Clamp M, Barton GJ (2009). Jalview Version 2–a multiple sequence alignment editor and analysis workbench. Bioinformatics.

[95] Lechat P, Hummel L, Rousseau S, Moszer I (2008). GenoList: an integrated environment for comparative analysis of microbial genomes. Nucleic acids research.

[96] Xayaphoummine A, Bucher T, Isambert H (2005). Kinefold web server for RNA/DNA folding path and structure prediction including pseudoknots and knots. Nucleic acids research.

[97] Zuker M (2003). Mfold web server for nucleic acid folding and hybridization prediction. Nucleic acids research.

[98] Lorenz R (2011). ViennaRNA Package 2.0. Algorithms Mol Biol.

[99] Diederichs K, Karplus PA (1997). Improved R-factors for diffraction data analysis in macromolecular crystallography. Nature structural biology.

