# Supplementary material for: Intermolecular base stacking mediates RNA-RNA interaction in a crystal structure of the RNA chaperone Hfq
Source: Sci Rep. 2017 Aug 29;7:9903. doi: 10.1038/s41598-017-10085-8 (PMC5575007; doi:10.1038/s41598-017-10085-8)
Supplement: Supplementary file 1 — Supplementary Information [file 41598_2017_10085_MOESM1_ESM.pdf]

Intermolecular base stacking mediates RNA-RNA interaction in a crystal structure of the RNA chaperone Hfq

Eike C. Schulz[1,2,5], Markus Seiler[1,6], Cecilia Zuliani[1], Franka Voigt[1,7], Vladimir Rybin[3], Vivian Pogenberg[2], Norbert Mücke[4], Matthias Wilmanns[2], Toby J. Gibson[1], and Orsolya Barabas[1*]

[1)] European Molecular Biology Laboratory, Structural and Computational Biology Unit, 69117 Heidelberg, Germany
[2)] European Molecular Biology Laboratory, Hamburg Outstation, Hamburg D-22603, Germany
[3)] European Molecular Biology Laboratory, Protein Expression and Purification Core Facility, 69117 Heidelberg, Germany
[4)] German Cancer Research Center, Division Biophysics of Macromolecules, Heidelberg D-69120, Germany
[5)] Present Address: Max Planck Institute for the Structure and Dynamics of Matter, Luruper Chaussee 149, 22761 Hamburg, Germany
[6)] Present Address: Buchmann Institute for Molecular Life Sciences, Max-von-Laue-Str. 15, 60438 Frankfurt a.M., Germany
[7)] Present Address: Friedrich Miescher Institute for Biomedical Research, Maulbeerstrasse 66, 4058 Basel, Switzerland

[*)] Correspondence and requests for materials should be addressed to O.B. (E-mail: barabas@embl.de)

Supplementary Information

Supplementary Tables:

Table S1: Summary of AUC data: Expected and measured sedimentation coefficients for the various samples.

SAMPLE	Expected [S]		Measured [S]
	monomer	dimer	
$Hfq72_6$	3.2*		3.1
A_{20}	1.3*		1.5
'AA0'	1.3**		1.3
$Hfq72_6$-A_{20}	3.9*	6.0*	5.1
$Hfq72_6$-'AA0'	3.9**		3.8
Hfq^{102}			3.5
fhlA			2.1
OxyS			2.0
Oxy0			n.d.
fhlA + OxyS			2.1
$Hfq102_6$-*fhlA*			4.5
$Hfq102_6$-OxyS			4.5
$Hfq102_6$-*fhlA*-OxyS			4.3 / 5.9
$Hfq102_6$-*fhlA*-Oxy0			4.4

*Calculated from the $Hfq72_6$-A_{20} crystal structure in program HYDROPRO 5a[1,2];
**Predicted based on the values calculated for the A_{20} RNA.

Table S2: Properties of candidate sRNAs

sRNA	sRNA length (nt)	$(ARN)_X$ motif coordinates	Distance to poly-U (nt)
**OxyS**	117	65-86	23
**OhsC**	77	21-43	24
**MicM**	84	26-43	33
**MgrR**	98	37-53	38
**RyjA**	140	86-100	32

Coordinates of sRNAs are given with respect to the first nucleotide

Table S3: sRNA and mRNA complementary regions

	sRNA			mRNA		
	Group	*coordinates*	*sequence*	*sequence*	*coordinates*	
OxyS	I	23;30	AACCCUUG	CAAGGGUU	34;41	***fhlA***
	II	64;75	GAAUAACUAAAG	CUAUAGUUAGUC	-57;-46	
	III	96;104	UCUCCAGGA	UCCUGGAAGA	-15;-6	
OhsC	I	2;20	UUGAGGGUGCAUGCUGCAC	GUGCAGCAUGCACCCUCAA	-94;-76	***shoB***
	II	38;44	GUAAAAC	GUUUUAC	52;58	
	III	50;56	UUCCUUA	UAAGGAA	-13;-7	
MicM	I	1;9	ACACCGUCG	CGCCGGUGU	45;53	***ybfM***
				UUUUUAUUUU	-62;-53	
	II	13;37	AAAGUGACGGCAUAAUAAUAAAAAA	UUUUUUAAUUUUUAU	-70;-56	
				UUUUUAUUUUUUCGCUGUUCACCUUU	-62;-37	
	III	33;45	AAAAAAUGAAA	UUUAUUUUUU	-60;-51	
	IV	42;56	AAUUCCUCUUUGACG	CGUCAAAGAGGAUU	-20;-7	
MicM	I	7;15	UCGCUUAAA	UUUAAGUGA	-124;-116	***dpiB***
	II	17;25	UGACGGCAU	AUCCGUCA	-39;-32	
	III	29;38	AAUAAAAAAA	UUUUAUUUAUU	-184;-174	
	IV	34;43	AAAAAUGAAA	UUUGCAUUUUU	28;38	
	V	46;57	CCUCUUUGACGG	CCGUCAAAGAGG	-37;-26	
	VI	62;68	AUAGCGA	UCGCUAU	-250;-244	
MgrR	I	15;23	GCAGGAAAA	UUUUCCCUGC	-68;-59	***eptB***
	II	39;46	AAGCAAAA	UUUUUGCUU	40;48	
	III	50;56	AAAUCUA	UAGAUUU	-99;-93	
	IV	52;68	AUCUAUCCAUGCAAGCA	UGUUUGCAUGAGAUACAU	-7;11	
	V	61;68	UGCAAGCA	UGCUUGCA	44;51	
MgrR	I	11;21	CAGUGCAGGAA	UUCCUGCCUG	-23;-14	***rsxE***
	II	21;31	AUGCCUGUU	AACAGGAU	-380;-373	
	III	32;39	AGCGUAAA	UUGUACGCU	-60;-54	
				GGCGGUGAA	-151;-145	
	VI	69;82	UUCACCGCCGGUUU	CCGCGCGGUG	-94;-87	
				ACCGGCG	-114;-108	
				AAACCGG	-369;-363	
	V	84;93	CUGGCGGUUU	AAACGCCAG	-248;-240	
RyjA	I	11;19	CGGAACC	GGUUCCG	-74;-68	***tig***
	II	25;34	CACCACGUGCUCG	CGAGCACUGUG	-29;-19	
	III	70;77	UGUACCAG	CUGGUAACA	-112;-100	
RyjA	I	45;54	GUUGACGUCG	CGAGGUCAAC	39;68	***nuoG***
	II	69;79	GUGUACCAGCG	CGCUGGUAAC	-55;-46	

Start and end coordinates of the complementary regions are indicated, separated by semicolon. Coordinates of sRNAs are given with respect to the first nucleotide and coordinates of mRNAs are given with respect to the first base of the translation. Roman numbers in the column labeled ‘Group’ indicate distinct complementary regions as shown in Figure 4.

Table S4: $(ARN)_X$ motif and RBS coordinates of selected mRNAs

mRNA	$(ARN)_X$ motif coordinates	$(ARN)_X$ motif sequences	$(ARN)_X$ motif distance (nt)	RBS coordinates	RBS sequence
fhlA	-75;-70<br>-9;-1	AAUAAA<br>AAGAACAAA	60	-11;-6	GGAAGAACAAA**AUG**
shoB	-77; -72<br>-12;+2	AAUAAC<br>AAGGAAAAACGAAU	59	-12;-8	AAGGAAAAACGA**AUG**
ybfM	-96;-86<br>-15;-3	AGUCAGCGAGA<br>AAAGAGGAUUAAC	70	-13;-8	AGAGGAUUAACCC**AUG**
dpiB	-78;-72<br>-10;+2	AAUUAAU<br>AAGGAAAACAAU	61	-10;-6	AAGGAAAACA**AUG**
eptB	-50;-42<br>+26;+34	AAAAGC<br>AGCAGAAGC	69	-13;-9	AGGGUUUGUUUGC**AUG**
rsxE	-10;-1<br>-144;-138	AAGAAAGA<br>AGAAUAAAGC	126	-12;-8	GGAGAAUAAAAC**GUG**
tig	-60;-54<br>-1;10	AACAAC<br>AGGUAACAAG	54	-11;-7	GAGGUAACAAG**AUG**
nuoG	-61;-54<br>-14;-1	AAAGAGC<br>AGAAAACUGGAAGC	40	-12;-8	GGAAGCAUGCUA**AUG**

Coordinates of mRNAs are given with respect to the first base of the translation.

Supplementary Figures:

Figure S1

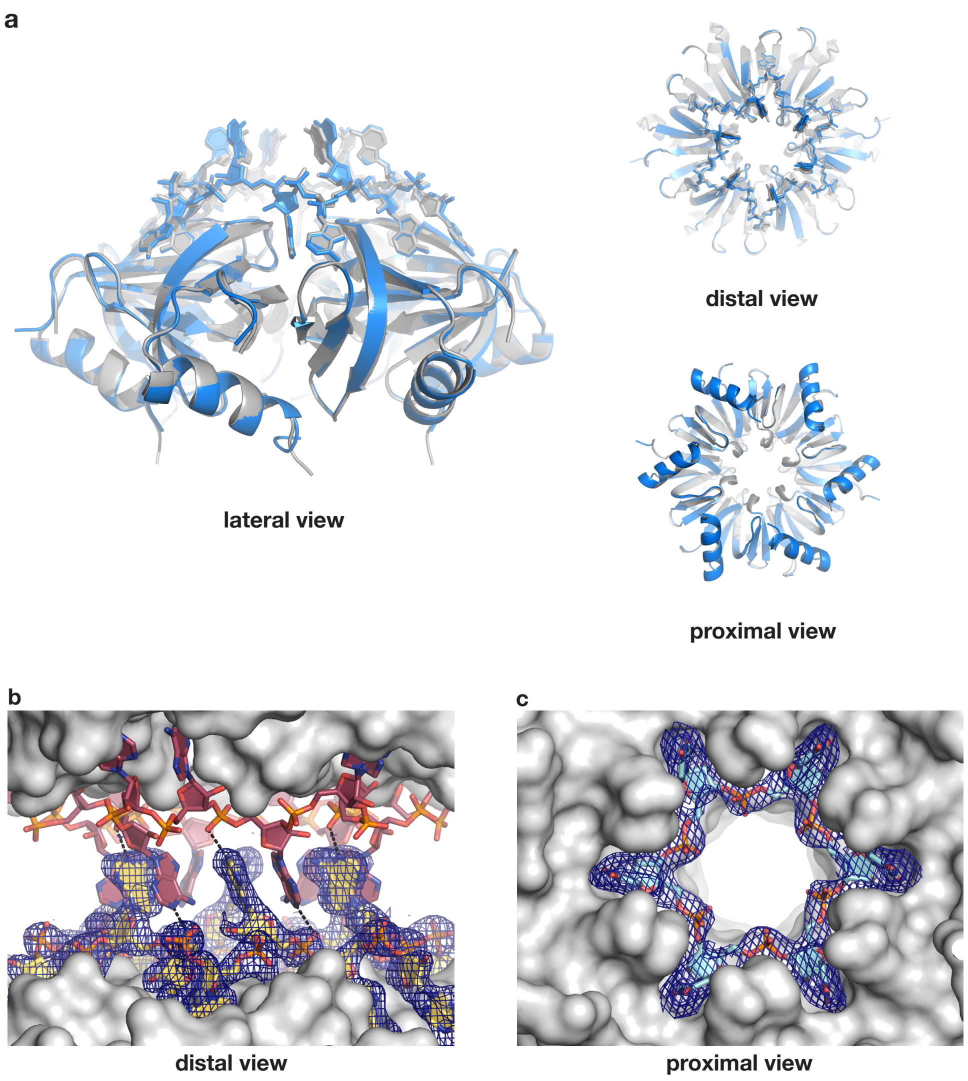

Figure S1: Base stacking of Hfq-A_{18} RNA complexes does not require alteration of the RNA configuration.

(a) Superposition of the base-stacked dimeric ($Hfq72_6$:A_{18})$_2$ complex structure with a previously reported *E.coli* Hfq-poly(A) crystal structure (PDB ID 3GIB[3]) shows only small differences; the N-site bases are tilted by 15º.

(b) 2Fo-Fc simulated annealed omit map contoured at the 1.0 σ level (blue mesh) showing clear density for the A_{18} RNA on the distal face of the Hfq72 hexamer in the $Hfq72_6$-A_{18} complex.

(c) 2Fo-Fc simulated annealed omit map contoured at the 1.0 σ level (blue mesh) at the proximal site suggests the presence of six uridine nucleotides. The binding geometry is practically identical with previously reported Hfq-poly(U) RNA complex structures[4,5]. Since no uridine containing RNA or nucleotides were included in the crystallization samples, this density probably originated from the cellular lysate or UTP contamination in the synthetic RNA probes. Maps were calculated in CNS[6,7].

Figure S2

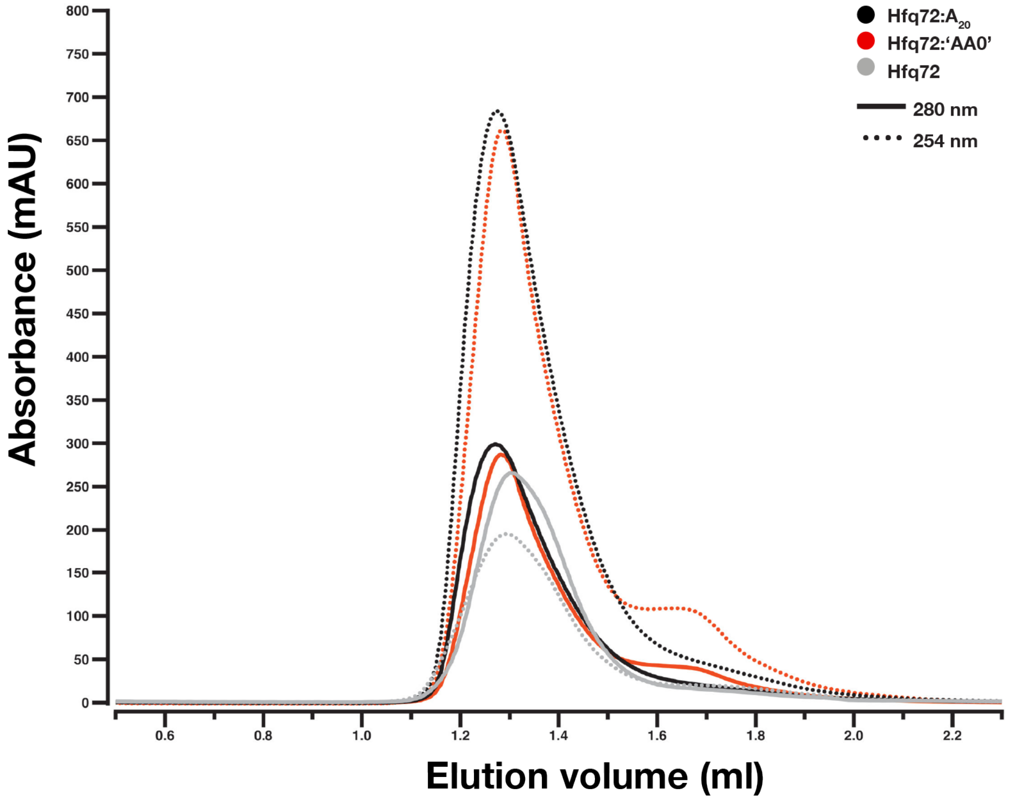

Figure S2: Introduction of an abasic nucleotide in 'AA0' RNA does not compromise Hfq binding
Analytical size exclusion chromatogram of the Hfq72-A_{20} RNA (black) complex, Hfq72-'AA0' RNA (red) complex, and Hfq72 alone (grey) on a Superdex S200 10/300 (GE Healthcare) column in AUC buffer (see Material and Methods). Solid lines show absorbance at 280 nm, while dashed lines show absorbance values measured at 254 nm. While RNA binding does not induce a significant difference in elution volume (1.30 ml and 1.27 ml for Hfq72 and Hfq72-A_{20} complex, respectively) consistent with the moderate change in molecular size (Mw = 48.7 kDa and 55.2 kDa for Hfq72 and Hfq72-A_{20} complex, respectively), the specific increase in 254 nm absorbance over the 280 nm absorbance in the complex samples, and the absence of a free RNA peak at 1.55 ml (data not shown) indicates similar levels of binding with both RNA molecules.

Figure S3

a **b**

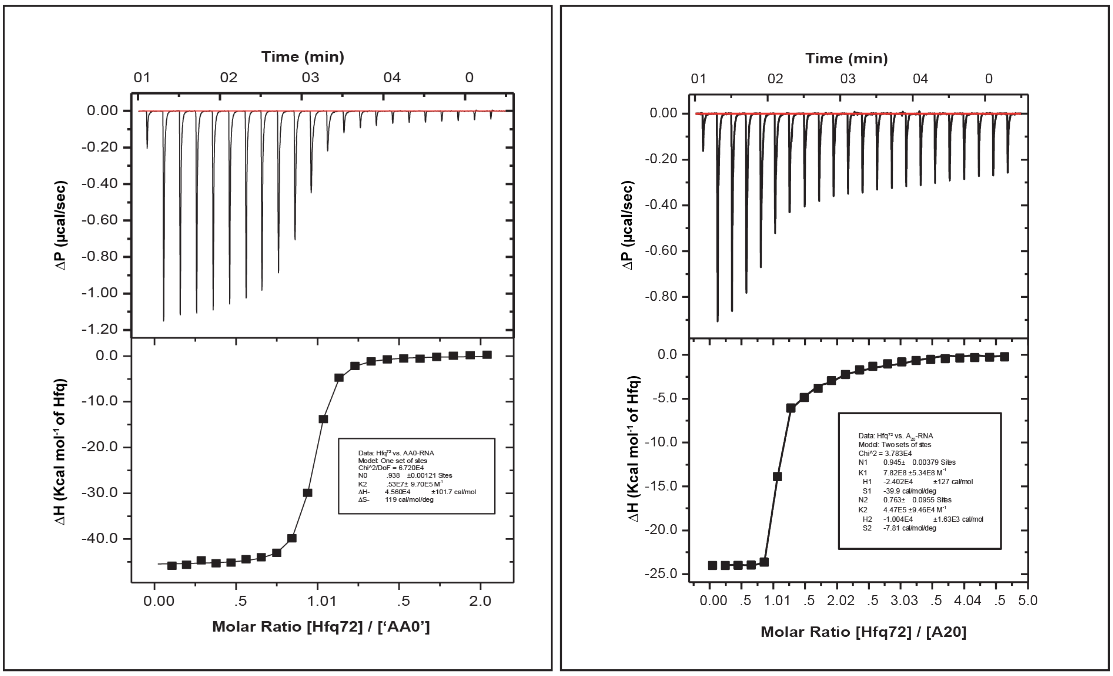

Hfq72 - ‘AA0’ **Hfq72 - A_{20}**

Figure S3: Isothermal titration calorimetry (ITC) of Hfq72 - ‘AA0’ and Hfq72 - A_{20} binding

(a) ITC data for Hfq72 - ‘AA0’ RNA binding shows a single binding event with an affinity of K_D = 40 nM. **(b)** ITC data for Hfq72 - A_{20} RNA binding indicating two consecutive binding events. The first binding event is very strong (K_D =1.3 nM), while the second binding event has a much lower affinity (K_D = 2.2 μM). Due to the large difference in the affinities, it was difficult to de-convolute the two binding events by ITC.

Figure S4

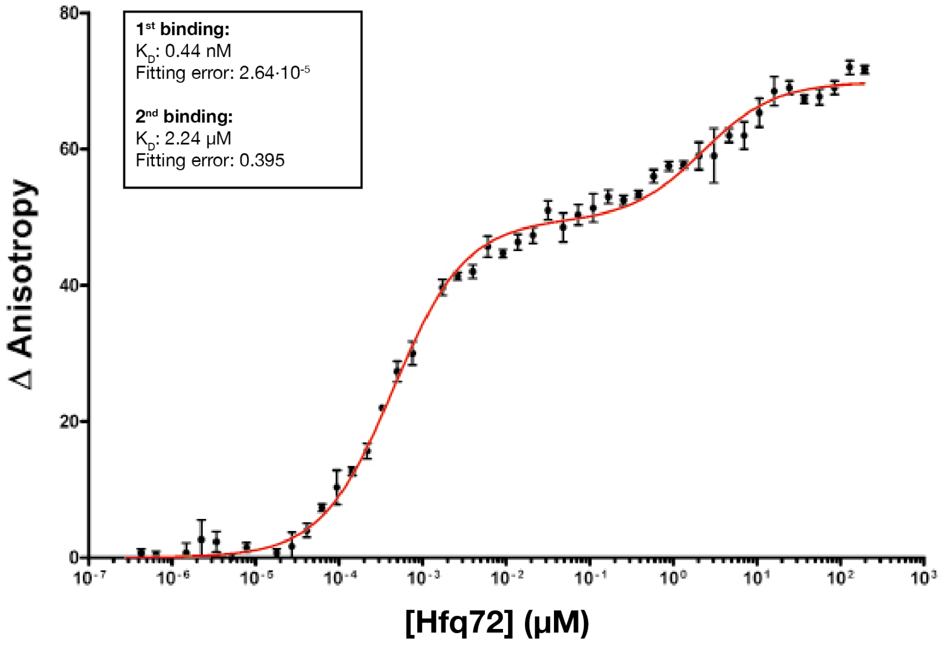

Figure S4: Fluorescence anisotropy of Hfq72 - A_{20} binding

Anisotropy difference plotted against Hfq concentration (µM) clearly showing a double sigmoidal curve indicating two consecutive binding events. In good agreement with the ITC data, the first binding has a K_D of 0.4 nM, while for the second binding event has a K_D of 2.2 µM. All data points were recorded in triplicates and error bars indicate the variation over these measurements.

Figure S5

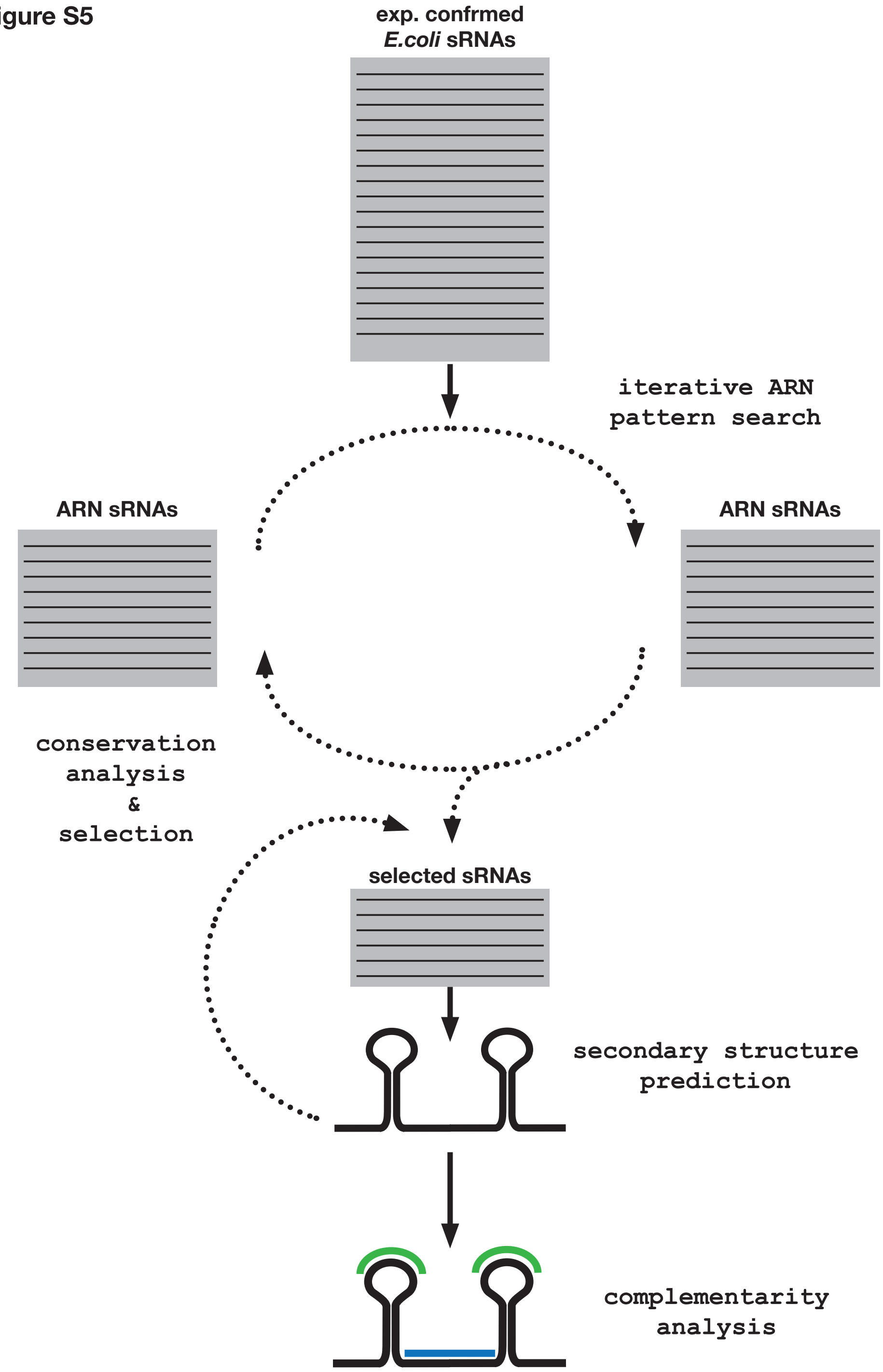

Figure S5: Bioinformatic analysis of sRNAs: The overall pipeline

An iterative $(ARN)_X$ pattern search (see details in Materials and Methods) in 67 experimentally confirmed *E.coli* sRNAs resulted in 25 sRNAs containing 1, 2 or 4 $(ARN)_X$ motifs. From this list, 5 candidate sRNAs were found to share a similar architecture of structural and functional elements.

Figure S6

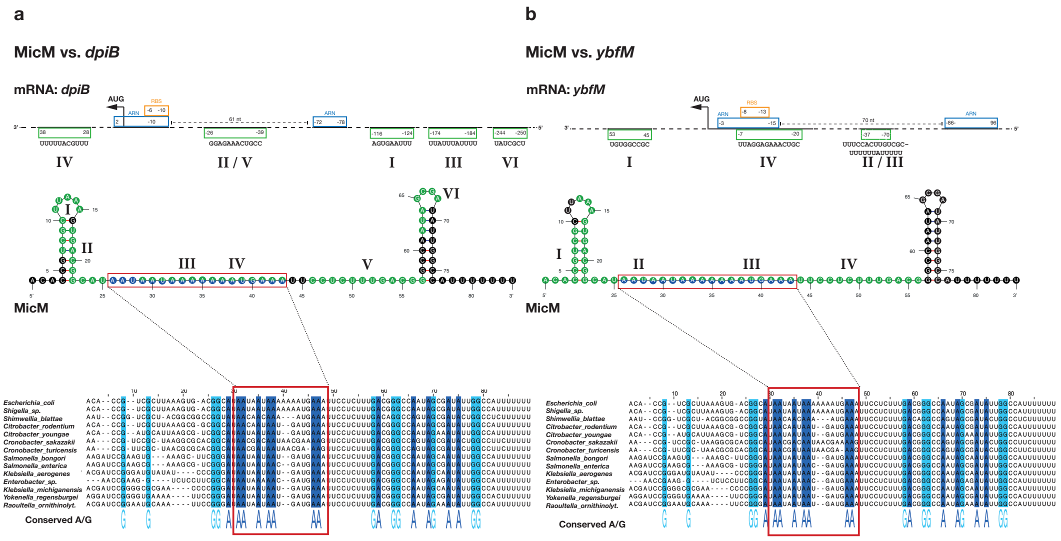

Figure S6: (ARN)$_X$ motifs and mRNA seed regions mapped onto the predicted secondary structure of MicM

The (ARN)$_X$ motif is found between two stem loops. Sequence alignment (bottom) for the (ARN)$_X$ motif containing region shows conservation of the motif among various species. The alignment contains sequences from the following species: *Escherichia coli* (NZ_KI544438.1), *Shigella sp.* (NZ_GG657387.1), *Shimwellia blattae* (NC_017910.1), *Citrobacter rodentium* (NC_013716.1), *Citrobacter youngae* (NZ_GG730299), *Cronobacter sakazakii* (NC_009778.1), *Cronobacter turicensis* (NC_013282.2), *Salmonella bongori* (NC_021870.1), *Salmonella enterica* (NZ_KE349762.1), *Klebsiella aerogenes* (NZ_KB911089.1), *Enterobacter sp.* (NZ_KI535662.1), *Klebsiella michiganensis* (NZ_JH603153.1), *Yokenella regensburgei* (NZ_JH417874.1), *Raoultella ornithinolytica* (NC_021066.1). Conserved A/G nucleotides are highlighted in blue/cyan. Seed regions for *dpiB* **(a)** and *ybfM* **(b)** pairing map to the stem loop structures (green dots). The topology of the targets mRNA *dpiB* **(a)** and *ybfM* **(b)** are shown above. (ARN)$_X$ regions (blue boxes) and seed regions (green boxes) are also marked. Roman numbers (consistent with Table S3) indicate the corresponding complementary sequences.

Figure S7

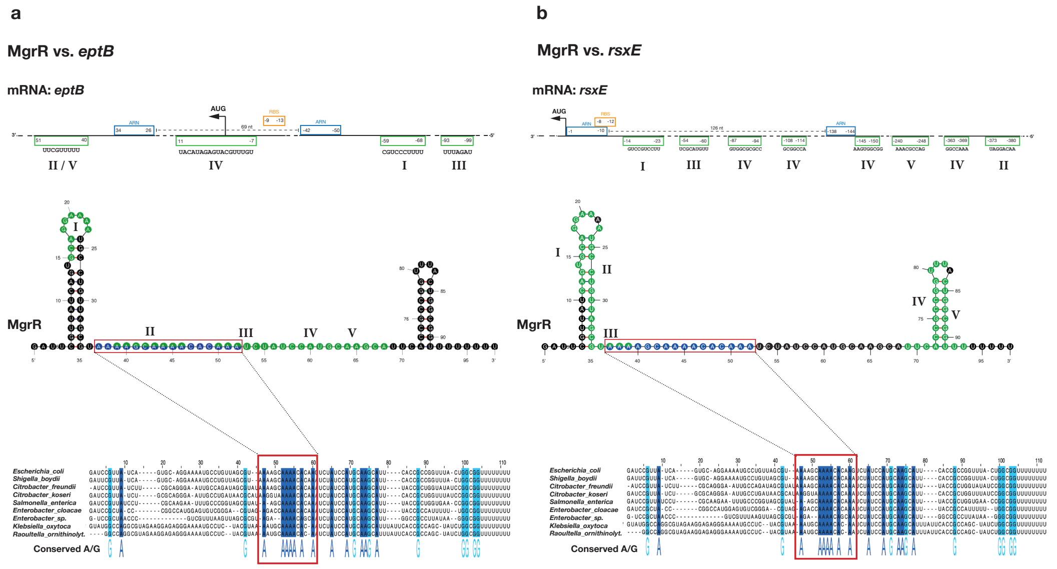

Figure S7: (ARN)$_X$ motifs and mRNA seed regions in MgrR

Structural and functional features of MgrR and its targets *eptB* **(a)** and *rsxE* **(b)** are shown as in Figure S6. The sequence alignment contains sequences of the following species: *Escherichia coli* (NZ_KE136969.1), *Shigella boydii* (NC_010658.1), *Citrobacter freundii* (NZ_JH414886.1), *Citrobacter koseri* (NC_009792.1), *Salmonella enterica* (NZ_KE350125.1), *Enterobacter cloacae* (NZ_KI535551.1), *Enterobacter sp.* (NC_021500.1), *Klebsiella oxytoca* (NZ_JH603146.1), *Raoultella ornithinolytica* (NC_021066).

Figure S8

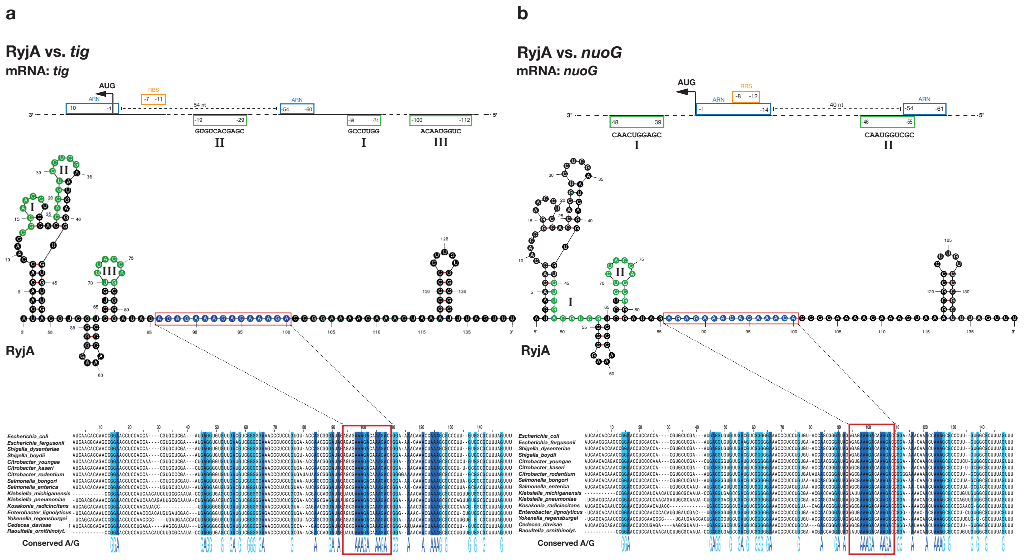

Figure S8: (ARN)$_X$ motifs and mRNA seed regions in RyjA

Structural and functional features of RyjA and its targets *tig* **(a)** and *nuoG* **(b)** are shown as in Figure S6. The sequence alignment contains sequences of the following species: *Escherichia coli* (NZ_KE701224.1), *Escherichia fergusonii* (NZ_CM001142.1), *Shigella dysenteriae* (NC_007606.1), *Shigella boydii* (NC_010658.1), *Citrobacter youngae* (NZ_GG730304.1), *Citrobacter koseri* (NC_009792.1), *Citrobacter rodentium* (NC_013716.1), *Salmonella bongori* (NC_021870.1), *Salmonella enterica* (NC_010067.1), *Klebsiella michiganensis* (NZ_JH603154.1), *Klebsiella pneumoniae* (NZ_KI535504.1), *Kosakonia radicincitans* (NZ_JH725436.1), *Enterobacter lignolyticus* (NC_014618.1), *Yokenella regensburgei* (NZ_JH417862.1), *Cedecea davisae* (NZ_KE161030.1), *Raoultella ornithinolytica* (NC_021066.1).

Figure S9

OhsC vs. *shoB*

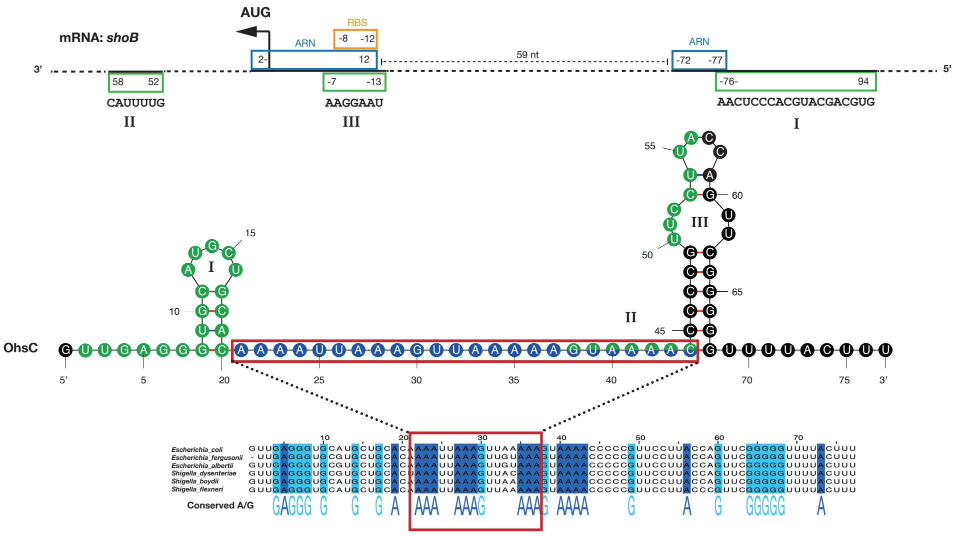

Figure S9: (ARN)$_X$ motifs and mRNA seed regions in OhsC

Structural and functional features of OhsC and its target *shoB* are shown as in Figure S6. The sequence alignment contains sequences of the following species: *Escherichia coli* (NZ_CP013952.1), *Escherichia fergusonii* (NZ_CM001142.1), *Escherichia albertii* (NZ_CH991859.1), *Shigella dysenteriae* (NC_007606.1), *Shigella boydii* (NC_010658.1), *Shigella flexneri* (NC_004337.2).

Figure S10

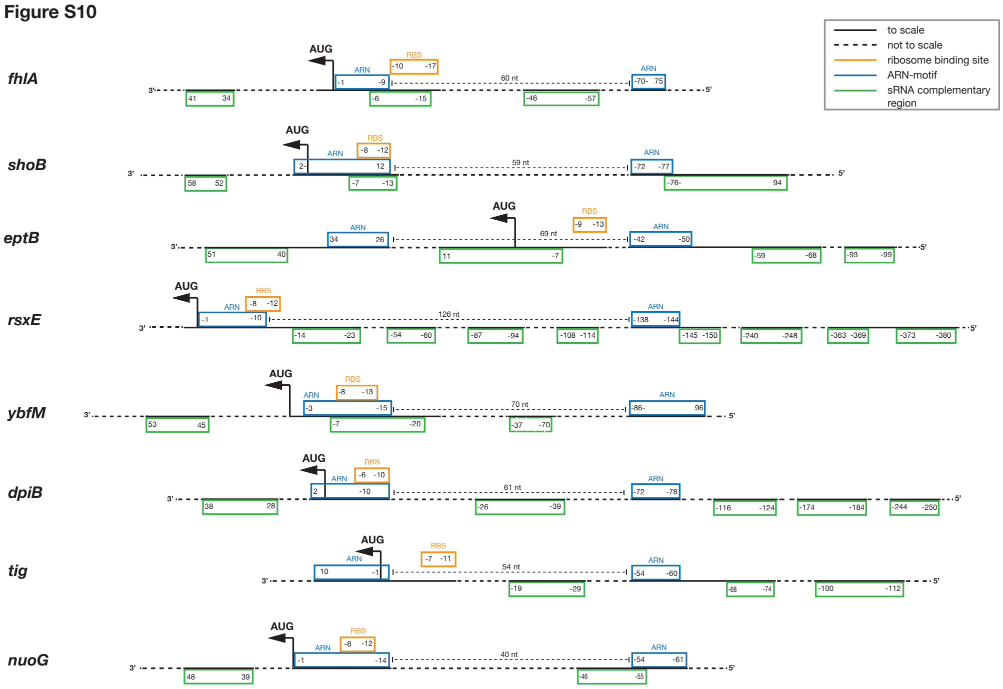

Figure S10: Topology of target mRNAs

mRNAs are shown in 3’ to 5’ direction, with their $(ARN)_X$ motifs aligned. Solid lines indicate distances to scale, dashed lines mark distances that are not displayed to scale. $(ARN)_X$ motifs (blue), ribosome binding sites (orange), and sRNA complementary regions (green) are shown. The position of the first base of translation is indicated with an arrow pointing in the direction of the CDS. The distance between the two separate $(ARN)_X$ regions are also marked.

References

1 Garcia De La Torre, J., Huertas, M. L. & Carrasco, B. Calculation of hydrodynamic properties of globular proteins from their atomic-level structure. *Biophys J* **78**, 719-730, doi:10.1016/S0006-3495(00)76630-6 (2000).

2 Ortega, A., Amoros, D. & Garcia de la Torre, J. Prediction of hydrodynamic and other solution properties of rigid proteins from atomic- and residue-level models. *Biophys J* **101**, 892-898, doi:10.1016/j.bpj.2011.06.046 (2011).

3 Link, T. M., Valentin-Hansen, P. & Brennan, R. G. Structure of Escherichia coli Hfq bound to polyriboadenylate RNA. *Proceedings of the National Academy of Sciences of the United States of America* **106**, 19292-19297, doi:10.1073/pnas.0908744106 (2009).

4 Schulz, E. C. & Barabas, O. Structure of an Escherichia coli Hfq:RNA complex at 0.97 A resolution. *Acta crystallographica. Section F, Structural biology communications* **70**, 1492-1497, doi:10.1107/S2053230X14020044 (2014).

5 Sauer, E. & Weichenrieder, O. Structural basis for RNA 3'-end recognition by Hfq. *Proceedings of the National Academy of Sciences of the United States of America* **108**, 13065-13070, doi:10.1073/pnas.1103420108 (2011).

6 Brunger, A. T. Version 1.2 of the Crystallography and NMR system. *Nature protocols* **2**, 2728-2733, doi:10.1038/nprot.2007.406 (2007).

7 Brunger, A. T. *et al.* Crystallography & NMR system: A new software suite for macromolecular structure determination. *Acta Crystallographica Section D-Biological Crystallography* **54**, 905-921 (1998).
